# Analysis and interpretation of transcriptomic data obtained from extended Warburg effect genes in patients with clear cell renal cell carcinoma

**DOI:** 10.18632/oncoscience.128

**Published:** 2015-02-17

**Authors:** Edward Sanders, Svenja Diehl

**Affiliations:** ^1^ Edward Sanders Scientific Consulting, Rue du Clos 33, Peseux, Switzerland; ^2^ Freelancer Bioinformatics, Germany

**Keywords:** Warburg effect, aerobic glycolysis, transcriptomics, clear cell renal cell carcinoma

## Abstract

**Background:**

Many cancers adopt a metabolism that is characterized by the well-known Warburg effect (aerobic glycolysis). Recently, numerous attempts have been made to treat cancer by targeting one or more gene products involved in this pathway without notable success. This work outlines a transcriptomic approach to identify genes that are highly perturbed in clear cell renal cell carcinoma (CCRCC).

**Methods:**

We developed a model of the extended Warburg effect and outlined the model using Cytoscape. Following this, gene expression fold changes (FCs) for tumor and adjacent normal tissue from patients with CCRCC (GSE6344) were mapped on to the network. Gene expression values with FCs of greater than two were considered as potential targets for treatment of CCRCC.

**Results:**

The Cytoscape network includes glycolysis, gluconeogenesis, the pentose phosphate pathway (PPP), the TCA cycle, the serine/glycine pathway, and partial glutaminolysis and fatty acid synthesis pathways. Gene expression FCs for nine of the 10 CCRCC patients in the GSE6344 data set were consistent with a shift to aerobic glycolysis. Genes involved in glycolysis and the synthesis and transport of lactate were over-expressed, as was the gene that codes for the kinase that inhibits the conversion of pyruvate to acetyl-CoA. Interestingly, genes that code for unique proteins involved in gluconeogenesis were strongly under-expressed as was also the case for the serine/glycine pathway. These latter two results suggest that the role attributed to the M2 isoform of pyruvate kinase (PKM2), frequently the principal isoform of PK present in cancer: i.e. causing a buildup of glucose metabolites that are shunted into branch pathways for synthesis of key biomolecules, may not be operative in CCRCC. The fact that there was no increase in the expression FC of any gene in the PPP is consistent with this hypothesis. Literature protein data generally support the transcriptomic findings.

**Conclusions:**

A number of key genes have been identified that could serve as valid targets for anti-cancer pharmaceutical agents. Genes that are highly over-expressed include ENO2, HK2, PFKP, SLC2A3, PDK1, and SLC16A1. Genes that are highly under-expressed include ALDOB, PKLR, PFKFB2, G6PC, PCK1, FBP1, PC, and SUCLG1.

## BACKGROUND

Approximately 90 years ago, the celebrated German biochemist and Nobel laureate Otto Warburg published his observations that cancer cells metabolized glucose more rapidly than normal cells and that the principal metabolic product was lactic acid (reviewed by Warburg [1]). This suggested that metabolism of glucose was halted at the terminal step of glycolysis; namely, the production of pyruvate, with pyruvate being converted into lactate. In most normal cells pyruvate is transported to the mitochondria where it is processed through the TCA cycle to complete the metabolism of glucose to water and CO_2_ with the accompanying production of 36 molecules of ATP per molecule of glucose. It is well known that oxygen is required for the TCA cycle; therefore, metabolism of glucose stops at the end of glycolysis, with concomitant lactate production, under hypoxic conditions. However, the metabolism of glucose in cancer cells produces large amounts of lactate even in the presence of ample oxygen – hence the designation aerobic glycolysis used interchangeably with the term “Warburg effect”.

With the intense focus on genetic changes in cancer brought about to a large extent by the discovery of oncogenes and tumor suppressor genes, there was little interest in the Warburg effect for many years. A 1976 comment by Sidney Weinhouse, cited by Gatenby and Gillies [2], is illustrative: “Since our perspectives have broadened over the years, the burning issue of glycolysis and respiration in cancer now flicker only dimly.” In the past 20 years, however, the Warburg effect has received increasing attention as playing a key role in cancer. This is clearly illustrated by a large number of recent reviews. Perhaps the most clear-cut proof that the altered metabolism of cancer cells is now in the mainstream of cancer research, however, is the inclusion of “reprogramming of energy metabolism” as an emerging hallmark of cancer by Hanahan and Weinberg [3].

It is currently well understood that the Warburg effect is not the cause of cancer. On the other hand, a relatively recent publication has shown that the genes involved in aerobic glycolysis are over-expressed in at least 24 different types of cancers corresponding to approximately 70% of all cancers [4]. Although specific genetic mutations are causally related to cancer, most cancers have been shown to have multiple mutations. For example, Ding et al. [5] sequenced 623 genes with known or potential relationships to cancer in 188 human lung adenocarcinomas and found more than 1000 somatic mutations across the samples. Even when they focused on genes that were frequently mutated, the number was still a non-trivial 26. Moreover, this analysis was restricted to adenocarcinoma, which constitutes no more than 50% of total lung cancer. Therefore, there is considerable logic in targeting a phenotype that is common to many cancers as opposed to a genotype that might be present in a limited percentage of a sub-type of a single cancer. Nevertheless, despite the wide-spread prevalence of the aerobic glycolytic phenotype in human cancers, drugs that have been tested focusing on potential targets known to be involved in this phenotype have exhibited only “modest effects” [6].

Part of the reason for the continuing lack of clinical success for potential anti-cancer compounds targeting the Warburg effect may be the fact that much of the current understanding of the steps involved has been derived from experiments in cultured cells, as was recently pointed out with respect to the role of pyruvate kinase M2 (PKM2) in cancer metabolism [7]. Although numerous studies have been published in the past 15 years comparing gene expression levels in tumor tissue to adjacent normal tissue, such studies have in general attempted to identify those genes that are highly over- or under-expressed in tumor tissue. Therefore, it is difficult to determine changes in a given pathway or network. As a consequence, we attempted to determine the transcriptomic changes that occur in an expanded network of aerobic glycolysis by first constructing such a network and then mapping gene expression values from a published data set comparing tumor tissue to adjacent normal tissue. The network consisted of glycolysis, the pentose phosphate pathway (PPP), the tricarboxylic acid cycle (TCA), gluconeogenesis, the serine/glycine pathway, and the initial steps of fatty acid synthesis and glutamine utilization, and it was constructed using Cytoscape (Supplementary Figure S1). The analysis of gene expression values was conducted on the most recent of three kidney cancer studies with data registered in the GEO that compared tumor to adjacent normal tissue for 10 patients all of whom were diagnosed with clear cell renal cell carcinoma (CCRCC) (GEO accession number GSE6344) [8.^9^].

CCRCC was chosen to investigate the Warburg effect because there was an extremely high probability that a large percentage of patients with CCRCC would exhibit this effect. The reason for this is that CCRCC, which comprises about 80% of all kidney cancers [10], is primarily caused by inactivation of the VHL gene either by mutation (50-60% of cases) or by methylation induced silencing (ca. 15% of cases) [11]. Inactivation of VHL leads to constitutive activation of HIF-1α, a protein that is generally only activated under hypoxic conditions. As a consequence, activation of HIF-1α via inactivation of the VHL gene has been referred to as pseudohypoxia [12,13], as opposed to true tumor hypoxia where increased levels of HIF-1α also play a major role [see, for example, 14]. HIF-1α reacts with HIF-1β (also known as ARNT) to form the dimer HIF-1, which migrates to the nucleus where it functions as a transcription factor for several hundred proteins [15]. Among those enzymes that are under HIF-1 transcriptional control are virtually all of the enzymes involved in aerobic glycolysis [15-19]. It is important to note that the clear-cut role played by pseudohypoxia caused by inactivation of VHL applies essentially only to CCRCC and not to most other cancers. The hereditary condition, VHL syndrome, is the result of one of several types of mutations in VHL. This condition, which occurs in roughly 1 in 36,000 live births, is responsible for 2-3% of CCRCC. However, with the exception of two rare types of malignancies, hemangioblastoma and pheochromocytoma, individuals with VHL syndrome are not at increased susceptibility to any other type of cancer [20].

The results of this analysis, discussed below, provide a detailed analysis of the difference of the extended metabolism of glucose between cancer tissue and normal tissue in CCRCC on the gene expression level.

## RESULTS AND DISCUSSION

**Figure 1:**
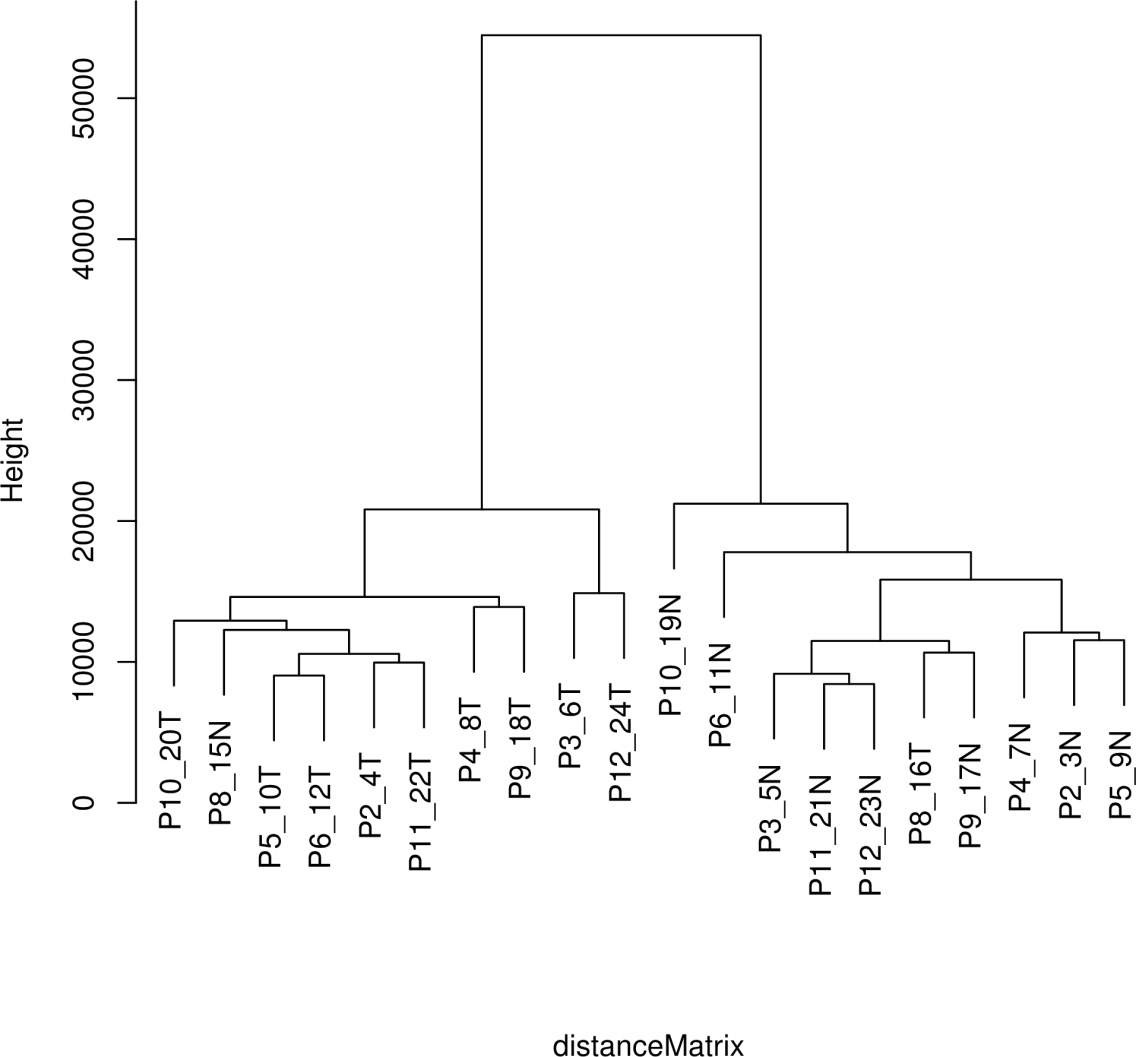
Hierarchical cluster analysis Distance matrix for the 10 CCRCC patients included in data set GSE6344 using the average linking clustering method based on Euclidean distances.

As indicated in the Background section, the analysis was conducted on the most recent of three kidney cancer studies with data registered in the GEO that compared tumor to adjacent normal tissue (GEO accession number GSE6344) [8,9]. This study analyzed data from 10 patients, all of whom had been diagnosed with CCRCC - five of these subjects were diagnosed with stage 1 cancer, and five were diagnosed with stage 2 cancer. Our analysis combined the data for all 10 patients. For each of the 10 patients, gene expression values for each gene in tumor tissue were matched to the corresponding values in adjacent normal tissue, and the gene expression ratios for the relevant genes were mapped on to the network (Supplementary Figure S1). The resulting data were first analyzed by hierarchical cluster analysis focusing only on the genes included in the network (Figure 1). Nine of the 10 patients gave results that clearly differentiated tumor tissue from normal tissue. Although patient 8 also demonstrated a difference between tumor and normal tissue, the results for tumor tissue matched the results for the normal tissue for the other nine patients and vice versa. As a consequence, the data for this patient were not analyzed further. Gene expression levels and fold changes (FCs) for all relevant genes in the GSE6344 data set are listed in Supplementary Table S1.

As previously noted, the network includes four major pathways; namely, glycolysis, gluconeogenesis, the pentose phosphate pathway (PPP), and the tricarboxylic acid (TCA) cycle. In addition, two short pathways are included, serine/glycine synthesis and utilization of fructose in glycolysis, as well as a portion of glutaminolysis and fatty acid synthesis. Each of these pathways will be discussed below.

### Glycolysis Pathway (Table 1) (Figure 2)

**Table 1:** Glycolytic genes over- or under-expressed by at least a factor of 2

Gene	FC Pat. 2	FC Pat. 3	FC Pat. 4	FC Pat. 5	FC Pat. 6	FC Pat. 9	FC Pat. 10	FC Pat. 11	FC Pat. 12	Average FC	p-value(from TDSIT)
**Over-expressed**											
*ENO2*	52.71	80.66	10.03	7.57	22.03	46.03	9.81	25.29	50.01	33.79	3.53E-06
*HK2*	55.80	10.81	6.19	4.68	23.24	4.68	30.80	8.66	43.06	20.88	3.52E-05
*PFKP*	13.30	5.84	4.69	6.85	8.86	8.85	6.74	9.76	7.11	8.00	4.23E-08
*SLC2A3*	5.89	6.52	4.55	5.89	3.10	11.90	13.48	3.05	3.37	6.42	1.30E-05
*ALDOC*	5.75	5.46	2.03	5.38	1.27	4.22	4.48	1.36	1.43	3.49	1.19E-03
*PKM*	6.06	2.32	2.41	2.06	3.18	3.15	1.22	3.06	5.23	3.19	1.72E-04
*PFKFB4*	9.27	1.39	1.90	2.32	2.33	3.15	1.86	1.80	2.96	3.00	1.00E-03
*SLC2A1*	2.15	1.49	1.64	1.29	1.80	3.99	1.17	3.72	3.38	2.29	1.58E-03
*HK1*	2.69	2.33	1.47	1.45	2.78	2.58	−1.02	2.68	3.50	2.27	5.82E-04
*ALDOA*	2.38	2.73	1.62	1.81	2.21	2.33	1.66	2.19	2.79	2.19	2.94E-06
*PGAM1*	2.95	2.11	1.56	2.23	2.08	2.04	2.19	2.03	1.75	2.10	1.51E-06
**Under-expressed**											
*ALDOB*	−82.38	−23.11	−35.40	−17.78	−10.01	−13.62	−135.83	−40.16	−658.50	−43.75	2.55E-05
*PKLR*	−10.99	A/A (1)	−12.13	A/A	A/A	A/A	A/A	−4.63	A/A	−8.51	1.13E-04
*PFKFB2*	−3.96	−5.78	−4.27	−8.35	−7.19	−8.58	−2.78	−3.39	−4.27	−5.02	2.18E-06
*VHL*	A/A	A/A	A/A	−3.43	−5.45	A/A	−3.06	A/A	−1.44	−3.01	3.96E-04

(1) T/N ratio not calculated; both tumor and normal tissue calls were absent

**Figure 2:**
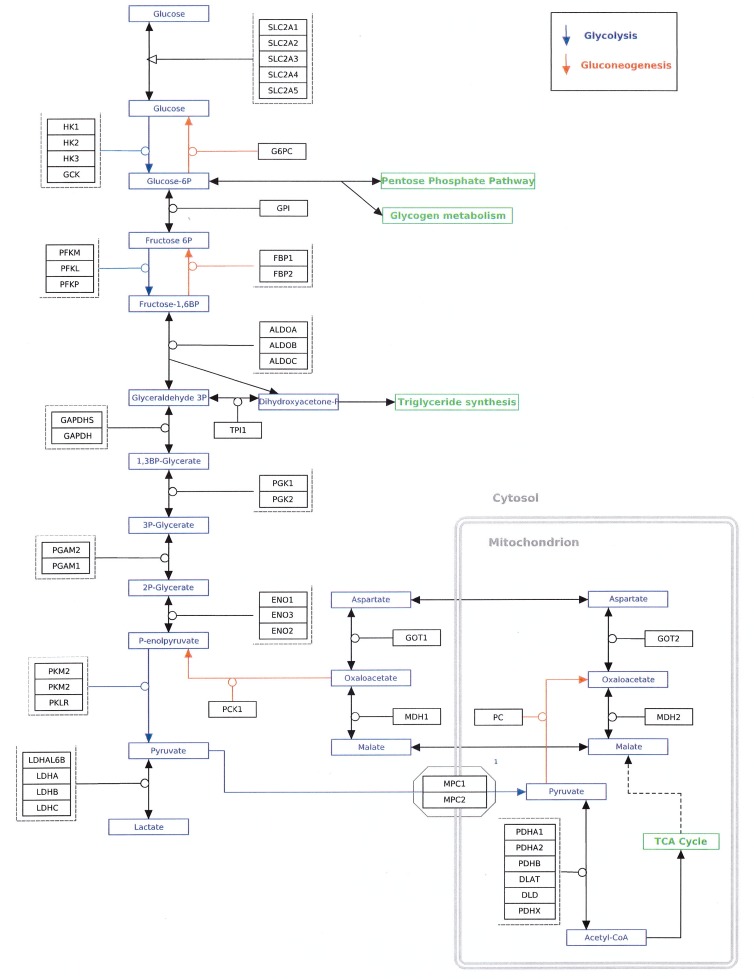
Glycolysis and gluconeogenesis pathways Enzymatic conversions designated by blue arrows are unique to glycolysis. Enzymatic conversions designated by red arrows are unique to gluconeogenesis. Enzymatic conversions designated by black arrows are reversible reactions common to both pathways. Taken from http://www.wikipathways.org/index.php/Pathway:WP534.

As noted, it is well known that *VHL* mutations play a major role in CCRCC. Inactivation of *VHL* results in constitutive expression of the HIF-1α protein resulting in a pseudohypoxic environment for the cancer tissue. It is impossible using gene expression data to confirm that *VHL* was actually mutated in the nine relevant patients. The involvement of *VHL* in this data set is supported, however, by the fact that it was under-expressed by a factor of 3 (p = 3.96E-04), although that result is based only on four of the nine patients in that the other five patients had absent calls for both tumor and adjacent normal tissues. Given the pseudohypoxic environment of the cancer tissue, it is not surprising that most genes involved in the glycolytic pathway were over- or under-expressed in the tumor tissue in a manner consistent with the literature dealing with the Warburg phenotype in cancer as well as with the known transcriptional effects of HIF-1. There are, however, some exceptions. This section will briefly discuss results for glycolytic genes that were consistent with the literature on the Warburg effect but will provide more detail for exceptions. For the vast majority of these genes the results are based on all nine patients unless otherwise specified.

#### Glucose Transporters

Of the five genes (*SLC2A1-5*) included in the network that code for the five glucose transport proteins GLUT1-5, both *SLC2A1* and *SLC2A3* were significantly over-expressed, with *SLC2A3* being up-regulated to a greater extent (FC = 6.42, p = 1.30E-05) than *SLC2A1* (FC = 2.29, p = 1.58E-03). Both *SLC2A1* and *SLC2A3* are known to be under transcriptional control of HIF-1 and are up-regulated in a number of cancers that exhibit the aerobic glycolytic phenotype [19]. In addition, GLUT1 protein levels have been reported to be increased in CCRCC [21].

#### Hexokinase (HK)

The *HK* genes code for protein hexokinases, which catalyze the phosphorylation of glucose to glucose-6-phosphate with ATP as the phosphate donor. Four different genes are known (*HK1/3*, *GCK*) each one of which codes for a different HK isoenzyme (I-IV). Isoenzymes I-II are characterized by a high affinity to their substrate. Isoenzyme IV, which is also known as glucokinase, has a low glucose affinity and is the predominant HK isoenzyme present in liver and pancreatic β cells. There is consistent evidence that the HK proteins, particularly HK II, are up-regulated in cancers that exhibit the Warburg phenotype [22]. The results of our gene expression analysis were quite consistent with this evidence. *HK1* was over-expressed by a factor of 2.27 (p = 5.82E-04), whereas *HK2* was very highly over-expressed, exhibiting a FC of close to 21 (p = 3.52E-05). All calls for both *HK3* and *GCK* were absent. Current theories suggest that HK II plays a key role in cancer because it binds to the voltage-dependent anion channel (VDAC) at the surface of the outer mitochondrial membrane. This binding has been reported to prevent the inhibition of hexokinase II by glucose-6-phosphate, thus allowing glycolytic flux to proceed at a higher rate. In addition, this binding possibly inhibits apoptosis [23]. There is also evidence that HK I can fulfill the same function [19,24,25]. Although gene expression levels of *HK2* showed a far greater increase than those for *HK1*, the gene expression levels of *HK1* in tumor tissue were greater in tumor tissue than those of *HK2*. Therefore, both genes may be playing a key role in aerobic glycolysis in CCRCC. There appears to be no evidence that either HK III or glucokinase bind to the VDAC, and levels of the genes that code for both enzymes are extremely low in both tumor and normal adjacent tissue as indicated by the fact that all calls were absent.

#### Glucose-6-phosphate isomerase (GPI)

The next step in the glycolytic pathway, the conversion of glucose-6-phosphate to fructose-6-phosphate, is catalyzed by the protein glucose-6-phosphate isomerase, which is coded for by the gene *GPI*. This gene was somewhat over-expressed, but the FC was well below a factor of 2 (FC = 1.51, p = 5.43E-03). Although *GPI* has been reported to be over-expressed in hypoxic tissues, it does not appear to be under transcriptional control of HIF-1 [19].

#### Fructose-6-phosphate kinase-1 (PFK)

Fructose-6-phosphate is converted to fructose-1,6-bisphosphate (F-1,6-BP) by the protein phosphofructokinase-1. There are three isoenzymes of this protein, PFKL (also known as PFK-B), PFKM (also known as PFKA and PFKX), and PFKP (also known as PFK-C and PFKF), and they are coded for by the three genes *PFKL*, *PFKM*, and *PFKP*, where L, M, and P represent liver, muscle, and platelet. The active forms of PFKL and PFKM are homotetramers, while the active form of PFKP can exist as a homotetramer or as a heterotetramer containing one or two PFKL moieties. Our gene expression analysis indicated that *PFKL* was essentially unchanged, whereas *PFKM* was slightly under-expressed, but by less than a factor of 2. Therefore, neither of these genes appears in Table 1. *PFKP*, on the other hand, was over-expressed by a factor of 8 (p = 4.23E-08). Although this result clearly indicates an increase in transcription of phosphofructokinase that would be expected for the aerobic glycolytic phenotype, it has been generally reported that only *PFKL* is under transcriptional control of HIF-1 [19]. However, recent literature has provided evidence that the *PFKP* gene is indeed over-expressed when HIF-1 is constitutively activated [26-28]. Whereas the PFK isoenzyme of differentiated tissues is mainly regulated by ATP:ADP:AMP ratios, which allows an optimal fine tuning of mitochondrial and glycolytic energy regeneration (Pasteur effect), in tumor cells PFK is mainly regulated by fructose-1,6-BP and fructose 2.6-BP (see below) [29]. There is unfortunately too little information in the literature to determine why the observed exclusive over-expression of *PFKP* appears to play a key role in the appearance of the Warburg phenotype in kidney cancer. A recent publication reports that over-expression of Krüppel-like factor 4 (KLF4) in four different breast cancer cell lines led to an increase in PFKP expression as well as an increase in glucose uptake and lactate production. In addition, a close correlation was found between KLF4 and PFKP levels in cells taken from breast tumors [30]. On the other hand, a more recent report indicates that KLF4 was clearly under-expressed in CCRCC tumor tissue compared to adjacent normal tissue. Furthermore, tumors in BALB/c nude mice generated by injection of 786-O renal cell adenocarcinoma cells carrying a KLF4 vector resulted in inhibition of tumor growth compared to a control. However, the observed inhibitory effect of this protein was reported to be through enhancement of the expression of p21*^WAF1/CIP1^* and reduction of cyclin D1 expression [31]. Clearly additional research into the role of PFKP in CCRCC might be of considerable value in utilizing this gene/protein as a potential target for anti-cancer drugs.

#### Fructose-6-phosphate kinase-2 (PFK2)

There is a branch point from the glycolytic pathway that involves an alternative phosphorylation of fructose-6-phosphate by one of four proteins, PFKFB1-4, coded for by four equivalent genes referred to collectively as *PFK2*. The product of this reaction is fructose-2,6-bisphosphate (F-2,6-BP). Each of these four proteins is bifunctional, containing a kinase moiety, which catalyzes the forward reaction, and a phosphatase moiety that catalyzes the reverse reaction [32]. As can be seen from Table 1, the gene *PFKFB4* was over-expressed by a factor of 3 (p = 1.00E-03), while *PFKFB2* was very highly under-expressed (FC = −5.02, p = 2.18E-06). The expression of both *PFKFB1* and *PFKFB3* was not changed by a factor of 2. *PFKFB3* was slightly over-expressed, whereas *PFKFB1* was slightly under-expressed; however this value is based on only the one patient that expressed a present call. As will be seen, these results are somewhat unexpected; therefore, it is of interest to discuss these four genes/proteins in some detail.

The first of the PFKFB proteins was isolated by Van Schaftingen and Hers in 1981 [33], which they designated as PFK2. They also presented evidence that suggested that not only did the product of this reaction, F-2,6-BP, enhance the activity of PFK1 (glycolysis), but it also inhibited the activity of fructose-1,6-bisphosphatase (FBPase-1/2) (gluconeogenesis). Further research has demonstrated that this protein is one of four isoenzymes that were renamed as 6-phosphofructo-2-kinase/fructose-2,6-bisphosphatase (PFKFB) thus specifying the bifunctional nature of these proteins. The early nomenclature for these proteins was based upon the organ in which each protein had been identified, but a convention designating the protein based on its coding gene was later adopted; namely, *PFKFB1-4*. Thus PFKFB1 is the designation for the protein first found in liver as well as a splice variety of this protein found in skeletal muscle. PFKFB2 became the designation for the heart isoenzyme, PFKFB3 for the brain/placenta isoenzyme, and PFKFB4 for the testis isoform. A number of splice varieties of PFKFB3 have been identified. For example the previous designation uPFK2 represents a splice variety as does iPFK2, where “u” stands for ubiquitous and “i” for inducible [34]. In addition, six splice varieties of PFKFB3 have been identified in human brain [35]. Research during the past 30 years has confirmed that F-2,6-BP is an extremely potent allosteric activator of PFK1, thus overcoming the inhibitory effect of ATP on PFK1 and accelerating glycolysis. There is some doubt, however, that F-2,6-BP inhibits the activity of FBPase; thus it may not slow down gluconeogenesis [36,37]. Nevertheless, a majority of articles published in the past ten years continue to state that PFKFB1-4 inhibits gluconeogenesis.

For approximately 20 years following the discovery of the PFKFB family of proteins, research was focused on their role in glucose metabolism in normal cells and organs, particularly the liver. About 10 years ago, however, the potential role of these proteins in cancer began to be explored. A number of reports established that PFKFB proteins were often up-regulated in cancer cell lines [reviewed recently for PFKFB3 by 38]. There were also a number of publications reporting over-expression of *PFKFB1-4* mRNA and/or proteins in human cancers, including colon, breast, ovarian, and thyroid carcinomas [39]; breast and colon malignant tumors [40]; human lung tumors [41]; and gastric cancers [42]. In addition, a number of reports were published during this period of time indicating that all of the *PFKFB1-4* genes were under transcriptional control of HIF-1 [42-46]. As early as 2004, Obach et al. [45] provided evidence that other proteins could transactivate at least *PFKFB3*, and indeed other such mediators have been identified, including TP53-induced regulator of glycolysis and apoptosis (TIGAR), although in this case the effect is a down-regulation [47]; IL-3 [48]; and progestin [49].

Research on the role of the *PFKFB1/4* genes and their corresponding proteins in cancer has primarily been focused on *PFKFB3*. The reason for this is that the protein coded for by this gene has been reported to have by far the highest kinase to phosphatase ratio (K/Pase) thus suggesting that an over-expression of *PFKFB3* in cancer, compared to other members of the family, would lead to increased F-2,6-BP and thus increased glycolytic flux. As early as 2002, it was suggested that constitutive expression of the protein PFKFB3 in several human cancer cell lines with high proliferation rates, coupled with its high K/Pase ratio, “could serve as an explanation for the high glycolytic rate in transformed cells even under normal oxygen tension (the Warburg effect).” [43]. A similar conclusion, to cite another example, was drawn by Calvo et al. [50], and this group suggested that the inhibition of *PFKFB3* might serve as an important target for anti-cancer drugs focused on inhibition of aerobic glycolysis. Indeed, some research has been conducted in an effort to develop compounds that can inhibit PFKFB3 with some success, although it would appear that none of these compounds has been evaluated clinically [51-53]. As a consequence, it was unexpected to find that there was no evidence of an over-expression of *PFKFB3* in the kidney cancer data, but rather an over-expression of *PFKFB4*.

As noted, it has been generally assumed that the PFKFB3 protein plays a key role in stimulating cancer cell proliferation because of its reported very high K/Pase ratio. Therefore, how might one explain the role being played by PFKFB4? Before exploring possible answers to that question, it is important to put current information on K/Pase ratios for the FPKFB1-4 proteins into perspective. There is one single publication that has reported the K/Pase ratio for human PFKFB3 (placental PFKFB), and the reported value was 710 [54]. There is also only one single publication that has reported the K/Pase ratio for human PFKFB4 (testis PFKFB), and the reported value was 0.9 [55]. Both of these results were obtained *in vitro*. Interestingly enough, it would appear that no measurements of the K/Pase ratio have been reported for human PFKFB1-2, although the ratio for these two proteins in other mammalian species has been determined [56]. Given the lack of human *in vivo* data for all four of these proteins, it is possible that further research may provide different results. In addition, the K/Pase ratio of all four PFKFB proteins can be modified by posttranslational modifications, particularly phosphorylation [see, for example, 34,57].

As it turns out there are two very recent publications that suggest that over-expression of *PFKFB4* rather than *PFKFB3* plays a role in the carcinogenic process in certain systems. Ros et al. [58] investigated three prostate cancer cell lines – DU145, PC3, and LNCaP. They found that silencing of *PFKFB4* with siRNA induced apoptosis in all three cell lines but observed no effect in a non-transformed cell line. In addition, no effect was observed when *PFKFB3* was silenced in any of the three transformed cell lines, although silencing of this gene in the non-transformed prostate epithelial cell line RWPE1 did result in a decrease in glycolytic rate. Moreover, activation of an inducible shRNA targeted to *PFKFB4* in PC3 cells transplanted into immunocompromised mice resulted in tumor regression. A paper by Goidts et al. [59] in the same year reported similar results for three glioma cancer stem like cell (CSC) lines. This group performed a loss-of-function screen using a shRNA library representing the entire human kinome and identified 46 proteins that are essential to the survival of these CSCs *in vitro*, one of which was PFKFB4. In order to assess the clinical relevance of these 46 proteins, a comparison was made of their mRNA expression among a series of astrocyte gliomas of different grades. Interestingly, primary glioblastoma patients whose tumors demonstrated *PFKFB4* mRNA expression above average had a highly significantly shorter survival time than patients with *PFKFB4* mRNA expression below average (p<0.0001). Although *PFKFB3* showed a slight increase in mRNA expression in primary glioblastomas relative to normal brain tissue (1.3), there was no correlation with *PFKFB3* expression levels and survival times.

Ros et al. hypothesized that the role of increased PFKFB4 is to lower the level of F-2,6-BP, which they base on the data cited above that PFKFB4 has a slightly higher phosphatase to kinase ratio [58]. Although this would decrease glycolytic flux, it would increase the level of glucose-6-phosphate, which in turn would increase flux through the PPP. This provides the cancer cell with increased reducing equivalents (NADPH), contributing to cancer cell survival through reduction of reactive oxygen species (ROS) [60] as well as providing key biomolecules required for cellular proliferation. On the other hand, Goidts et al. [59] present a completely different explanation. They report that knockdown of *PFKFB4* in one of their CSC cell lines (NCH421k) reduced levels of both lactate and ATP leading to cell death via apoptosis. They hypothesized that the resulting decrease in ATP as a result of knockdown of *PFKFB4* increases the AMP/ATP ratio, which in turn activates AMP-activated protein kinase leading to an inhibition of mTOR followed by apoptosis. This result suggests that the PFKFB4 protein is increasing glycolytic flux in these cells, a conclusion that is essentially diametrically opposed to that of Ros et al [58]. It is not at all impossible that both of the explanations cited above are correct, in that the role of PFKFB4 may be completely different in different cellular contexts.

It is impossible at this point to explain the role of over-expression of *PFKFB4* in the nine CCRCC patients that we analyzed. In addition, there is essentially no literature dealing with the role of any of the PFKFB1-4-isozymes in kidney cancer. It may well be that PFKFB4 could be playing a role in addition to its effect on glycolytic flux. For example, Yalcin et al. [61] recently reported evidence that PFKFB3 is functioning in a manner that is distinct from its glycolytic role; namely, nuclear translocation followed by activation of several key cell cycle proteins including Cdk-1, Cdc25C, and cyclin D3. Further complicating the picture is the five-fold under-expression of *PFKFB2*, for which there appears to be no precedent. All that can be concluded is that attempting to treat CCRCC by targeting PFKFB3 is likely to be unprofitable, whereas targeting PFKFB4 may be efficacious. As stated by Dang [62] in his commentary on the Ros et al. [58] publication, “The challenge for targeting cancer cell metabolism is being able to precisely profile the cancer cell metabolome according to the type of cancer and to identify the metabolic Achilles' heel.”

#### Aldolase (ALDO)

The following step in the glycolytic pathway is the conversion of F-1,6-BP to the two trioses, glyceraldehyde-3-phosphate (G3P) and dihydroxyacetone phosphate (DHAP) catalyzed by aldolase. Three isoenzymes of aldolase, which are encoded by the genes *ALDOA-C*, have been described. Aldolase A is expressed primarily in muscle and erythrocytes. Aldolase B is characteristically expressed in liver, kidney and the small intestine, while aldolase C is mainly expressed in neuronal tissues. Both *ALDOA* and *ALDOC* were over-expressed in the nine CCRC tumors evaluated (FC = 2.19, p = 2.94E-06; FC = 3.49, p = 1.19E-03, respectively). Increases in aldolase A and C through the transcriptional activity of HIF-1 on *ALDOA and ALDOC* has been frequently reported [19]. Therefore, the over-expression of these two genes in kidney cancer is to be expected. In addition there have been a number of proteomic and histochemical studies of renal cancer tissues that confirm the up-regulation of both aldolase A and C [63-66] *ALDOB*, on the other hand, was highly significantly under-expressed (FC = −43.75, p = 2.55E-05). In that this under-expression is most likely a consequence of the role that aldolase B plays in gluconeogenesis, further discussion will be deferred until this pathway is addressed.

#### Triosephosphate isomerase (TPI1)

The two three-carbon metabolites, G3P and DHAP, produced from the action of aldolase on F-1,6-BP can be interconverted by the protein triosephosphate isomerase (TPI) coded for by the gene *TPI1*. This gene has been reported to be under transcriptional control of HIF-1 and to be over-expressed in cancer [19]. Although there was a consistent over-expression of *TPI1* in this data set, it did not achieve a two-fold increase in expression (FC = 1.63, p = 5.31E-05). Lastly, it should be mentioned that DHAP can be utilized as the starting point for triglyceride and phospholipid synthesis (Supplementary Figure S1), but this pathway was not explored.

#### Glyceraldehyde-3-phosphate dehydrogenase (GAPDH)

Glyceraldehyde-3-phosphate dehydrogenase (GAPDH), coded for by the *GAPDH* gene, catalyzes the conversion of glyceraldehyde-3-P to glycerate-1,3-P2 using NAD and phosphate as co-substrates. There are reports in the literature that indicate that the *GAPDH* gene is not under transcriptional control of HIF-1 and that the protein is not up-regulated in cancer [67,68]. Our analysis is consistent with these reports in that the *GAPDH* gene expression level was virtually unchanged.

#### Phosphoglycerate kinase (PGK1)

The enzyme PGK1, coded for by the gene *PGK1*, catalyzes the conversion of 1,3-bisphosphoglycerate to 3-phosphoglycerate. The gene has been reported to be over-expressed in cancer and under transcriptional control of HIF-1 [19]. In this data set, however, the expression of *PGK1* was found to be unchanged (FC = −1.09). 3-Phosphoglycerate is the point of initiation of a pathway that branches from glycolysis and ultimately serves to synthesize serine and glycine. This pathway will be discussed in detail below.

#### Phosphoglycerate mutase (PGAM)

The enzyme phosphoglycerate mutase (PGAM) catalyzes the isomerization of 3-phosphoglycerate to 2-phosphoglycerate. There are two monomeric isoforms of this enzyme, phosphoglycerate mutase 1 (PGAM 1, also known as PGAM B) and PGAM 2 (also known as PGAM M), coded for by the genes *PGAM1/2*. The active protein consists of homo- or heterodimers of PGAM B and M with muscle and sperm cells primarily expressing MM homodimers. It has been reported that *PGAM1* is under transcriptional control of HIF-1 and is elevated in cancer [19]. Our analysis was in agreement with this, showing over-expression of *PGAM1* (FC = 2.10, p = 1.51E-06). In addition, Unwin et al. [66] reported that the protein PGAM 1 (PGAM B) was increased in RCC tissue compared to adjacent normal tissue. Curiously, *PGAM2* was found to be somewhat under-expressed. Although the ratio was slightly greater than 2 (−2.18), all calls were absent meaning that the result is not particularly reliable.

#### Enolase (ENO)

The next step in the glycolytic pathway involves the conversion of 2-phosphoglycerate to phosphoenolpyruvate (PEP). This reaction is catalyzed by the protein enolase, which consists of homo- or heterodimers of enolase α, enolase γ, and enolase β, which are coded for by the genes *ENO1/3*. As can be seen from Table 1, the gene with the greatest increase in expression in the glycolysis pathway was *ENO2* (FC = 33.79, p = 3.53E-06). *ENO2* codes for the protein γ-enolase and is expressed almost entirely in mature neurons, neural-related cells, and neuroendocrine (NE) cells [69]. As a consequence, it is often referred to as neuron specific enolase. It has been known for quite some time that γ-enolase is frequently increased in NE tumors compared to adjacent normal tissue. For example, the increase in γ-enolase, normalized for the change in α-enolase level, was 33 in neuroblastoma tumors and 16 in small cell lung cancer (SCLC) [70]. Gamma-enolase is detectable in serum, and a moderate increase in serum levels of γ-enolase in individuals with various types of renal cell cancers was first reported by Takashi et al. in 1989 [70]. This finding has been confirmed by other researchers investigating γ-enolase levels in serum [71-73] and in interstitial fluid [74], as well as quantification of γ-enolase by immunohistochemistry [75,76] and proteomic analysis comparing CCRCC tissue to normal adjacent tissue [77,78]. These results provide support to the gene expression analysis reported above. Despite our reported highly significant increase in the expression of *ENO2* in CCRCC tumor tissue coupled with the proteomic confirmation with respect to increased levels of γ-enolase, the evidence suggests that CCRCC is not of neuroendocrine origin. Neuroendocrine kidney tumors do exist, but they are extremely rare [79].

There is no evidence to suggest that *ENO2* or its corresponding protein, γ-enolase, has any function beyond its role in glycolysis; namely, the conversion of 2-phosphoglycerate to PEP. As a consequence, it appears unlikely that the over-expression of ENO2 in tumor tissue could be an indication of an alternative pathway that might provide a competitive advantage to proliferating cancer cells. However, a recent paper by Vander Heiden et al. [80] provides evidence for a possible role for the observed over-expression. These authors hypothesized that the fact that PKM2 is significantly less reactive than PKM1 would result in an accumulation of PEP in cancer cells where PKM2 has replaced PKM1. In investigating the possible result of such an accumulation, both *in vitro* and in cell lysates, an alternative synthesis of pyruvate was identified – one that neither utilizes PK nor produces ATP. This reaction occurs by the transfer of a phosphate group from PEP, thus producing pyruvate, to a 25-kD protein, which the authors identified as being PGAM 1. In addition, they report that the reaction occurs only when PKM2 is present. Further work determined that the site of phosphorylation is histidine (His 11). Given that phosphorylation of His 11 is required for the enzymatic activity of PGAM 1, increasing the extent of phosphorylation of His 11 leads to increased PGAM 1 activity.

Vander Heiden et al. [80] proposed two possible explanations for the importance of this alternate use of PEP in cancer. The first is that it prevents excess production of ATP thus obviating feedback inhibition of PFK-1. The second is that it increases the level of activated PGAM 1, which may generate a positive feedback loop, given that the PGAM 1 catalyzed inter-conversion of 3-phosphoglycerate and 2-phosphoglycerate is reversible. Both of these explanations suggest that there may be a branch point in glycolysis downstream of F-1,6-BP and upstream of 2-phosphoglycerate that produces biomolecules essential for cellular proliferation. The following two years saw the appearance of three publications that identified such a branch point; namely, the production of serine and glycine from 3-phosphoglycerate. Of particular interest is that three different approaches were used to establish the increase in production of serine and glycine as playing an important role in cancer; namely, functional genomics [81], metabolomics [82], and flux balance modeling [83]. In addition Vié et al.[84] demonstrated that *PSAT1*, the gene that codes for the second enzyme in the serine biosynthesis pathway branching from 3-phosphoglycerate, is over-expressed in tumor tissue samples obtained from 29 colorectal cancer (CRC) patients, and that the level of *PSAT1* mRNA is inversely correlated with response to conventional chemotherapy for CRC.

As will be discussed below no conclusion can be drawn from the kidney cancer data that the nine CCRCC patients who clearly exhibit the aerobic glycolytic phenotype have replaced PKM1 by PKM2. A very reasonable hypothesis would be that if PEP does not accumulate because PKM1 does not appear to be replaced by PKM2, *ENO2* could be significantly over-expressed in order to produce the pool of PEP required to cause increased serine and glycine synthesis from 3-phosphoglycerate. However, inspection of Table 2, which provides gene expression values for the serine/glycine pathway using the kidney cancer CCRCC data, indicates that this is not the case. This pathway will be discussed in more detail below.

**Table 2:** Gene expression FCs for genes Involved in the serine/glycine synthesis pathway

Gene	FC Pat. 2	FC Pat. 3	FC Pat. 4	FC Pat. 5	FC Pat. 6	FC Pat. 9	FC Pat. 10	FC Pat. 11	FC Pat. 12	Average FC	p-value (from TDSIT)
*PSAT1*	−28.85	−36.43	−16.73	−61.88	−100.44	−20.74	−18.36	−6.66	−3.38	−21.39	2.20E-05
*PHGDH*	−1.12	−12.13	−2.48	−2.38	−2.84	−7.58	−2.15	−3.30	−16.53	−3.89	1.78E-03
*SHMT1*	−4.25	−2.72	−3.48	−2.50	−8.51	−2.20	−2.20	−4.71	−3.71	−3.47	2.73E-05
*PSPH*	−1.22	2.00	1.12	1.72	−1.26	−2.65	−1.04	−1.27	−3.05	−1.17	4.45E-01

Currently, it appears that there is no obvious explanation for the highly increased expression of *ENO2* in these patients. Nevertheless, the data would strongly suggest that this gene/protein would make an excellent target for anti-cancer drugs designed to interfere with aerobic glycolysis in CCRCC.

#### Pyruvate Kinase (PK)

The enzyme PK catalyzes the dephosphorylation of PEP to pyruvate, the last step of glycolysis, and is responsible for net ATP production within glycolysis. This production of ATP is independent of oxygen supply, unlike production of ATP in the TCA cycle, thus allowing tissues to survive under anaerobic conditions. Four different isoenzymes of PK are expressed depending on the different metabolic demands of the tissues in which they are expressed; namely, PKM1, PKL, PKR, and PKM2. PKM1 has the highest affinity for its substrate, PEP, is not allosterically regulated, and is the characteristic PK isoenzyme of cells and tissues with high energy demand such as muscle and brain. The L isoenzyme has the lowest affinity for PEP and is expressed in tissues that have high rates of gluconeogenesis such as liver, kidney, and intestine. Pyruvate kinase type R is expressed in erythrocytes. The isoenzyme M2 is expressed in some differentiated tissues, such as lung and adipose tissue, as well as in all highly proliferating cells including normal proliferating cells, embryonic cells, adult stem cells, and tumor cells in particular [85].

All four of the PK isoenzymes exist as tetramers in their active state. In contrast to the other PK isoenzymes, PKM2 may also occur in a dimeric form. Kinetic characterizations revealed that under physiological conditions the tetrameric form of PKM2 is highly active, whereas the dimeric form is nearly inactive [85,86]. The ratio of active tetramer to inactive dimer is not fixed but changes in response to both activating and deactivating factors, which allows an optimal adaption of metabolism to different conditions, e.g., nutrient supply [87]. An important allosteric activator of PKM2 is the glycolytic metabolite F-1,6-BP. High levels of F-1,6-BP induce the association of two dimers to the highly active tetrameric form [88-93]. In addition, the amino acid L-serine has also been reported to allosterically activate PKM2 through conversion of the dimer to the tetramer [87,94,95]. In tumor cells, the inactive dimeric form was found to be predominant due to direct interaction with different oncoproteins, including the E7 oncoprotein of the human papillomavirus type 16, as well as several tyrosine kinases, [87,96-98]. Recently, Christofk et al. [99] reported that phosphotyrosine proteins interact with the PKM2:F-1,6-BP complex to displace F-1,6-BP thus allowing the tetramer to revert to the dimer. Several other mechanisms have been proposed including tyrosine phosphorylation, lysine acetylation, cysteine oxidation, and prolyl hydroxylation [100]. The amount of dimeric PKM2 protein in plasma was shown to correlate with staging in different cancers including breast cancer [101,102], lung cancer [103,104], cervical carcinoma [105], and melanoma [106]. In addition, levels of PKM2 in stool correlate with staging of colorectal cancer and have been used for colorectal cancer screening as has been clearly shown by a meta-analysis of 17 studies [107].

The four PK isoenzymes are coded for by two genes; namely *PKLR* and *PKM* (also known as *PKM2*). The *PKLR* gene codes for both PKL and PKR under the control of two different tissue specific promoters [108]. *PKLR* was strongly under-expressed in the CCRCC tissue (FC = −8.51, p = 1.13E-04) (Table 1), although this result was based on only three patients. This suggests that either PKL or PKR or both play a less important role in glucose metabolism in these tumors than in normal tissue. There is a report of positive staining for PKL protein in various renal cancers, including CCRCC [109]; however, this enzyme was also present in normal renal tissue and a quantitative comparison between normal and tumor tissue could not be made. *PKM*, on the other hand, was over-expressed in cancer tissue (FC = 3.19, p = 1.72E-04) (Table 1). From the data available, however, it was not possible to discriminate whether PKM1 or PKM2, is up-regulated, since these two proteins are splice varieties coded for by the same gene. Exon 9 of the *PKM* gene is transcribed in PKM1, whereas exon 10 is transcribed in PKM2. Recently it has been reported that four isoforms of two proteins are responsible for splicing of exon 10 into pyruvate kinase thus leading to the transcription of PKM2. These four proteins are coded for by the genes *HNRNPA1*, *HNRNPA2B1*, *PTB1*, and *PTB2*. This report also provided evidence indicating that transcription of these proteins is up-regulated by c-Myc [110]. A later publication by the same group provided references indicating that these proteins are over-expressed in various cancers [111]. The genes that code for the four regulators of *PKM* splicing were also included in the expanded glycolysis network (Supplementary Figure S1). Although all were over-expressed, none of them were over-expressed by a factor of 2 with *PTB2* having the largest FC (1.50, p = 0.01). FCs for the remaining three genes were: 1.38 for *HNRNPA2B1* (p = 0.007), 1.19 for *PTBP1* (p = 0.02), and 1.05 for *HNRNPA1* (p = 0.6). This result could suggest that there is no shift from PKM1 in normal tissue to PKM2 in CCRCC tissue; however, this would seem to be unlikely. This is despite the fact that a recent report supports such a result. Bluemlein et al. [112] conducted an absolute quantification of the PKM1 and PKM2 isoforms in 25 human malignant cancers, 6 benign oncocytomas, tissue-matched controls, and several cell lines. In all cases it was shown that PKM2 was the prominent isoform in all cancer samples and cell lines. However, they report that PKM2 was also the principal isoform in the matched tissue samples.

Notwithstanding the Bluemlein et al. [112] result, there are numerous studies that have reported significantly higher levels of PKM2 isoenzyme in renal cancer. Wechsel et al. [113] reported that the level of PKM2 as determined by immunohistochemistry was significantly increased in 40 RCC patients compared to 39 controls (p = 0.0001). Oremek et al. [114] used an ELISA-based assay to compare levels of dimeric PKM2 in plasma from 116 RCC patients compared to 42 patients suffering from nephritis. Once again, there was a highly statistically significant difference in PKM2 levels between the two groups of subjects. Similar results were reported by Hegele et al. [115]. Perroud et al. [77] reported a ratio of 14.7 for PKM2/PKM1 comparing CCRCC tissue to normal adjacent kidney tissue using proteomics. A more recent proteomic study also reported an increase of pyruvate kinase in CCRCC tumor tissue compared to normal adjacent tissue; however, this study utilized a single patient and the isoform of pyruvate kinase was not specified [116]. On the other hand, it should be noted that Unwin et al. [66] published an early proteomic study that reported increased levels of PKM2 in RCC tumor tissue compared to normal kidney tissue in six patients but a more pronounced increase in PKM1 levels in these six patients (2.1-3.5- and 2.4-14.8-fold increase, respectively).

Based on the reports cited above, there would seem to be little question that levels of PKM2 protein are elevated in CCRCC tumor tissue compared to normal adjacent tissue. Therefore, it would appear that basing a conclusion as to the lack of a shift from PKM1 to PKM2 on the absence of over-expression of the four genes that code for the proteins responsible for the alternate splicing is not warranted. One possible finding that could possibly contribute to resolving this issue is that of Nisman et al. [117], who reported that there was a strong positive correlation with levels of dimeric PKM2 and tumor grade (p = 0.001). Given that the nine subjects investigated in this analysis exhibited either stage 1 or 2 CCRCC, it is possible that the levels of PKM2 were only slightly elevated in the tumor tissue.

The importance of the isoform of PKM present in kidney cancer rests on the fact thata there is virtually universal agreement as to the role that PKM2 is playing in cancer. This is that the lowered activity of PKM2 as compared to PKM1 provides cancer cells with a proliferative advantage by forcing a buildup of metabolites that can be used to synthesize key biomolecules such as nucleotides and amino acids [85,99,100,118]. Specifically, production of CO and GDP, which constitutes the second PEP would accumulate forcing glycolysis to proceed in the reverse direction, thereby increasing the levels of proteins that constitute branch points to the glycolytic pathway. To investigate the possible role of the production of such biomolecules in CCRCC, three specific pathways were investigated; namely, gluconeogenesis, the reverse of glycolysis; and two pathways that branch off from glycolysis; namely the serine/glycine pathway and the pentose phosphate pathway (PPP).

### Gluconeogenesis

Gluconeogenesis is an anabolic pathway leading to the synthesis of glucose, which is then used to synthesize glycogen (Figure 2). Although the liver is generally considered to be the principal organ involved in human gluconeogenesis, it is well known that the kidney plays a key role as well [119]. Gluconeogenesis is essentially the reverse of glycolysis. Therefore, if a key aspect of the metabolism of glucose involves reversal of glycolysis, at least upstream of PEP because of the reduced activity of PKM2 compared to PKM1, one would expect to see an active gluconeogenesis pathway. With the exception of the reactions catalyzed by hexokinase, phosphofructokinase, and pyruvate kinase, the enzymes involved in glycolysis catalyze both the forward and reverse reactions. As a consequence, no conclusions can be drawn from expression data for these genes as to which direction the reaction is proceeding with the exception, as will be seen, of the aldolase genes and proteins. However, in the three reactions catalyzed by the enzymes mentioned above, different proteins are utilized in glycolysis and gluconeogenesis, and useful information can be obtained from gene expression values. These steps will be discussed below, and the data are tabulated in Table 3.

**Table 3:** Gene expression FCs for gluconeogenesis and fructose metabolism genes

Gene	FC Pat. 2	FC Pat. 3	FC Pat. 4	FC Pat. 5	FC Pat. 6	FC Pat. 9	FC Pat. 10	FC Pat. 11	FC Pat. 12	Average FC	p-value (from TDSIT)
*G6PC*	−31.66	−19.47	−304.97	−7.26	−21.32	−69.94	−99.07	−147.53	−127.90	−53.84	8.38E-06
*ALDOB*	−82.38	−23.11	−35.40	−17.78	−10.01	−13.62	−135.83	−40.16	−658.50	−43.75	2.55E-05
*PCK1 (1)*	−11.87	−10.50	−15.28	−2.88	−13.13	−5.94	−4.52	−21.55	−1086.66	−8.96	1.67E-05
*FBP1*	−11.74	−12.17	−7.21	−7.52	−6.73	−9.92	−3.97	−9.55	−11.61	−8.48	1.04E-07
*PC*	−8.81	A/A (2)	A/A	−19.56	−5.46	−7.18	A/A	−5.34	−7.81	−8.10	2.18E-05
*PCK2*	−8.92	−8.76	−7.94	−6.23	−10.36	−7.33	−2.02	−8.56	−12.93	−7.39	3.24E-06
*KHK*	−1.88	−5.93	−2.61	−2.04	−2.91	−3.37	−1.42	−3.07	−3.57	−2.75	8.61E-05
*MDH1*	−1.32	−2.53	−1.45	−1.98	−2.63	−2.19	−1.38	−1.59	−1.87	−1.83	1.14E-04
*G6PC2*	−1.61	−1.45	−1.20	A/A	A/A	−1.08	A/A	−1.37	−1.09	−1.29	1.47E-02
*G6PC3*	−1.84	−1.45	−1.37	−1.25	−1.78	−1.42	1.13	−1.03	1.26	−1.27	4.17E-02
*SLC2A5*	1.42	1.34	−4.36	1.39	−1.16	1.08	−1.51	−1.24	−1.19	−1.16	4.41E-01

(1) The T/N value for patient 12 was not included

(2) T/N ratio not calculated; both tumor and normal tissue calls were absent

#### Pyruvate Carboxylase (PC) and Phosphoenolpyruvate carboxykinase (PCK)

The conversion of PEP to pyruvate by any of the PK isoenzymes is not reversible, and it requires at least two steps to reconvert pyruvate to PEP. Following the transport of pyruvate into the mitochondria, it can be converted to oxaloacetate in the first step by the protein pyruvate carboxylase (PC) [120], which is coded for by the *PC* gene. The active form of the PC protein is a homotetramer located in the mitochondrial matrix. *PC* was very highly under-expressed in the CCRCC tumor tissue with a FC of −8.10 (p = 2.18E-05), although this result could be based on only six patients. Two genes, phosphoenolpyruvate carboxykinase 1/2 (*PCK1/2*), code for the two proteins PEPCK-C and PEPCK-M, respectively. These two proteins are responsible for the ALDOA conversion of oxaloacetate to PEP, with the concomitant production of Co2 and GDP, which constitutes the second step of gluconeogenesis. The protein PEPCK-M is located in the mitochondria, whereas PEPCK-C is located in the cytosol, and the distribution of these two proteins differs markedly as a function of species. For example, rabbits, guinea pigs, and avian species express almost 100% PEPCK-M in the liver, although there is evidence that chicken kidney does express some PEPCK-C [121,122]. In these species the second step of the conversion of pyruvate to PEP, the conversion of oxaloacetate to PEP, is catalyzed by PEPCK-M, and the PEP is exported from the mitochondria [123]. On the other hand in rats and mice 90-95% of PEPCK activity is contributed by PEPCK-C, at least in the liver [124]. In such cases, the oxaloacetate formed in the first step must be converted to either malate or aspartate, which is, in turn, transported from the mitochondria to the cytosol. Reconversion of either of these two 4-carbon metabolites to oxaloacetate is then followed by production of PEP in the cytosol catalyzed by PEPCK-C [125,126]. Therefore, three steps are required. Mitochondrial and cytosolic hepatic human PEPCK are known to be about equally divided [124], and PEPCK-C has also been shown to be present in human kidney [127]. Therefore, it must be assumed that both pathways are active. This assumption is of no real importance, however, since both of *PCK* genes were very strongly under-expressed in CCRCC tumor tissue compared to normal tissue of the nine subjects examined, with a FC of –8.96 (p = 1.67E-05) for *PCK1* and a FC of −7.39 (p = 3.24E-06) for *PCK2*. Therefore, the conversion of pyruvate to PEP was strongly inhibited in CCRCC. This finding, however, provides no useful information as to the putative role of PKM2 on the build-up of glycolysis metabolites, since it reflects only the synthesis of additional PEP

#### Aldolase B (ALDOB)

As noted in the section on glycolysis, both *ALDOA and ALDOC* were over-expressed, whereas *ALDOB* was very strongly under-expressed. The expression of this gene in tumor tissue was about 2% of the expression in normal tissue (p = 2.55E-05) (Tables 1 and 3). The strong decrease in *ALDOB* expression in CCRCC tissue compared to normal tissue has been reported by other investigators [9,128,129], and similar results have been published for the level of the ALDOB protein [63,77]. An explanation for the very significant under-expression of *ALDOB* compared to a significant over-expression of *ALDOA* and C is that the ALDOA and C proteins appear to be much more effective in catalyzing the forward reaction (glycolysis), whereas ALDOB is much more effective in catalyzing the reverse reaction (gluconeogenesis). ALDOB has a 10-fold lower K_m_ for G3P and DHAP than does ALDOA [130], while cleaves F-1,6-BP about 25 times more rapidly than does ALDOB based on the comparative k_cat_ values [131]. Of particular interest are the results of Yanez et al. [132], who reported that ALDOB co-localizes with fructose-1,6-bisphosphatase (Fru-1,6-Pase) and PEPCK, both of which are key gluconeogenic proteins, in the proximal tubule cells of normal renal tissue, whereas ALDOA co-localizes with PK in the distal tubules and collecting ducts. These results led Yanez et al. [132] to propose that ALDOB participates primarily in the gluconeogenesis pathway, while ALDOA participates in glycolysis. As a consequence, the strong under-expression of *ALDOB* in CCRCC suggests that reverse of the aldolase step of glycolysis; namely, the synthesis of F-1,6-BP from the condensation of G3P and DHAP, does not take place in CCRCC tumor tissue.

#### ALDOB and Fructose Metabolism – SLC2A5 and KHK

The ALDOB protein plays another key role in the extended glycolysis pathway; namely, the conversion of fructose-derived fructose-1-phosphate (Fru-1-P) to a mixture of G3P and DHAP [133] (Supplementary Figure S1). Interestingly, mutations in *ALDOB* are responsible for a rare but potentially fatal condition known as hereditary fructose intolerance [133-135]. The very strong under-expression of *ALDOB* in the nine CCRCC tumors investigated would suggest that fructose does not contribute to the formation of the two metabolites cited above, which could be used either in glycolysis or gluconeogenesis. To test this hypothesis we first examined the kidney cancer data focusing on the genes coding for proteins linking fructose to glycolysis/gluconeogenesis. These genes are *SLC2A5*, which codes for GLUT5, a key fructose transporter; *KHK*, the gene coding for ketohexokinase, the protein that converts fructose to Fru-1-P; and *ALDOB*. It should be noted that the initial products from the action of the ALDOB protein on fructose-1-phosphate are glyceraldehyde and DHAP. The enzyme triokinase then catalyzes the conversion of glyceraldehyde to G3P. However, the gene that codes for this protein appears to be unknown. The expression values for these genes are listed in Table 3. As can be seen, two of the three are significantly under-expressed in tumor tissue. The results for *ALDOB* have already been mentioned. *KHK* was under-expressed by a factor of 2.75 (p = 8.61E-05). *SLC2A5*, however, was essentially unchanged (FC = −1.16, p = 4.41E-01). Secondly, we surveyed the literature to determine if other investigators had observed under-expression of any of these genes or down-regulation of the corresponding proteins in renal cancer. At least two groups of researchers have reported that KHK enzyme activity [136] and protein levels [136,137] are reduced in CCRCC as compared to normal tissue. On the other hand, two recent papers from a single working group reported an increase in *SLC2A5* in CCRCC and, thereby, an increase in the fructose transporter GLUT5 [138,139]. This result is not in agreement with the kidney cancer data analyzed herein. Moreover, the more recent of these two papers provides unreferenced statements suggesting that their data is consistent with the fact that both the enzymes FBPase and G6Pase are increased significantly in CCRCC. As will be seen below, this statement is not only inconsistent with the gene expression data for the 9 CCRCC patients analyzed in this work but is also inconsistent with enzyme activity levels cited by other researchers. This issue may perhaps cast some doubt on the conclusions drawn in these two papers.

#### Fructose 1,6-phosphatase (FBP)

The fourth specific gene involved in gluconeogenesis is *FBP1/2*, which codes for the protein FBPase-1/2. This protein catalyzes the conversion of F-1,6-BP to Fru-6-P, the reverse of the glycolytic reaction catalyzed by PFK1. *FBP1* was highly significantly under-expressed in CRCC (FC = −8.48, p = 1.04E-07). No meaningful FC could be calculated for *FBP2*, since all calls were absent. Inspection indicates, however, that this gene was slightly under-expressed. Several investigators have reported that protein levels and activities of FBPase were significantly reduced in CCRCC compared to normal kidney levels [66,109,140] confirming inhibition of this step of gluconeogenesis. A very recent paper provides compelling further evidence [141]. These authors reported that FBPase-1 was inhibited at the level of protein accumulation in almost 100% of more than 200 CCRCC tumors examined compared to normal kidney tissue.

#### Glucose-6-phosphatase (G6PC)

The last specific gene in the gluconeogenesis pathway is *G6PC*. There are three forms of this gene, *G6PC*, *G6PC2*, and *G6PC3*, which code for the 3 isoenyzmes G6Pase, G6Pase 2, and G6Pase 3. These three proteins catalyze the conversion of glucose-6-phosphate to glucose, the reverse of the first step of glycolysis catalyzed by hexokinase. *G6PC* was highly significantly under-expressed in the nine CRCC samples, with a fold-change of −53.84 (p = 8.38E-06). The genes that code for the other two isoenzymes are only slightly under-expressed (*G6PC2*, FC = −1.29, p = 1.47E-02, based on 6 patients; *G6PC3*, FC = −1.27, p = 4.17E-02). It has been previously reported that levels of G6PC enzyme are strongly reduced in CCRCC [10,140], a finding that tends to confirm this analysis.

To conclude this section, the gene expression data utilized in this study clearly demonstrate that gluconeogenesis does not appear to be functional in the nine patients with CCRCC that exhibited the Warburg effect. Moreover, this conclusion is well-supported by published reports on gluconeogenesis protein levels in CCRCC. This suggests that either PKM1 is not replaced by PKM2, as indicated by the data on the lack of over-expression of the four proteins responsible for the alternate splicing of the PKM proteins, or that despite the lower reactivity of PKM2, there is no reversal of glycolytic flux. However, the fact that gluconeogenesis is strongly reduced in CCRCC does not necessarily rule out that replacement of PKM1 by the less active PKM2 could result in increasing levels of metabolites up-stream of phosphoenolpyruvate. Since the gluconeogenesis “bottleneck” does not occur until ALDOB, mass action effects could indeed allow the build-up of metabolites until this point is reached. Therefore, increases in 3-phosphoglycerate, the branch point for serine/glycine synthesis, and glyceraldehyde-3-phosphate, the branch point for the non-oxidative branch of the PPP, would still be possible. On the other hand, the expression values of *ALDOB* and *FBP* essentially rule out increased flux through the oxidative branch of the PPP. As a consequence, gene expression data for both of these pathways were investigated.

### Serine/Glycine Synthesis Pathway – Phosphoglycerate dehydrogenase (PHGDH), phosphoserine aminotransferase 1 (PSAT1), phosphoserine transferase (PSPH), and serine hydroxymethyltransferase (SHMT1)

There are four steps involved in the conversion of 3-phosphoglycerate to serine and glycine. The first step is catalyzed by the protein D-3-phosphoglycerate dehydrogenase (PHGDH) coded for by the gene *PHDGH*. The product of this reaction, 3-phosphohydroxypyruvate, is converted to 3-phosphoserine, with the concomitant conversion of glutamate to 2-oxoglutarate, by the enzyme phosphoserine aminotransferase (PSAT) coded for by the gene *PSAT1*. 3-Phosphoserine is then dephosphorylated to serine by the protein phosphoserine phosphatase (PSPH), coded for by the gene *PSPH*. Lastly, serine is converted to glycine by the protein serine hydroxymethyltransferase (SHMT cytosolic) coded for by the gene *SHMT1*. Gene expression values for the nine patients that exhibited the Warburg effect are listed in Table 2. As can be seen, three of these four genes were strongly under-expressed with *PSAT1* exhibiting a FC of −21.39 (p = 2.20E-05), *PHGDH* exhibiting a FC of −3.89 (p = 1.78E-03), and *SHMT1* a FC of −3.47 (p = 1.03E-05). The fourth gene, *PSPH*, showed essentially no change in activity between normal kidney tissue and tumor tissue.

No corresponding data were found in the literature regarding expression levels of any of these four proteins in human CCRCC. The only vaguely pertinent published data relate to a comparison of the protein levels for PSAT1 and SHMT1 in a renal carcinoma transplanted in a rat and normal kidney tissue in the rat [142]. This study reported a small decrease in the activity of PSAT1, comparing tumor to normal tissue, but a slight increase in activity for SHMT1. There are, however, recent data on three of these four proteins in other cancer tissue. Toyama et al. [143] reported that PSAT1 was highly significantly up-regulated in clear cell ovarian cancer, but only slightly up-regulated in endometrial ovarian cancer. No change in PSAT1 level was observed for either mucinous or serous ovarian cancer. Up-regulation of PHGDH protein has n reported in both human melanoma and breast cancer. Increased levels of this protein in melanoma appear in part to be caused by an increase in *PHGDH* copy number; however, protein increase in breast cancer is associated with an increase in *PHGDH* mRNA. It is interesting to note that the increase of PHGDH was associated with distinct types of breast cancer [82]. Increased levels of SHMT were reported in plasma of patients with breast or ovarian cancer [144]. More recently, levels of SHMT were found to be increased in thyroid follicular adenoma as compared to normal tissue. Interestingly, there was an almost perfect linear correlation with increases in SHMT and PKM2 protein levels [145].

Taken together, in the nine renal tumors analyzed three of the four genes involved were significantly down-regulated. This finding is inconsistent with a reversal of the glycolytic pathway at the level of PEP or is inconsistent with the role of such a reversal being responsible for the increased synthesis of at least serine and glycine. Evidence cited above for protein expression levels of the first three proteins in the pathway suggest that flux through this pathway is increased in some other types of cancers. This could indicate that the reversal of glycolysis does occur in other types of cancer possibly caused by the lower reactivity of PKM2 compared to PKM1.

### Pentose Phosphate Pathway (PPP) (Figure 3)

**Figure 3:**
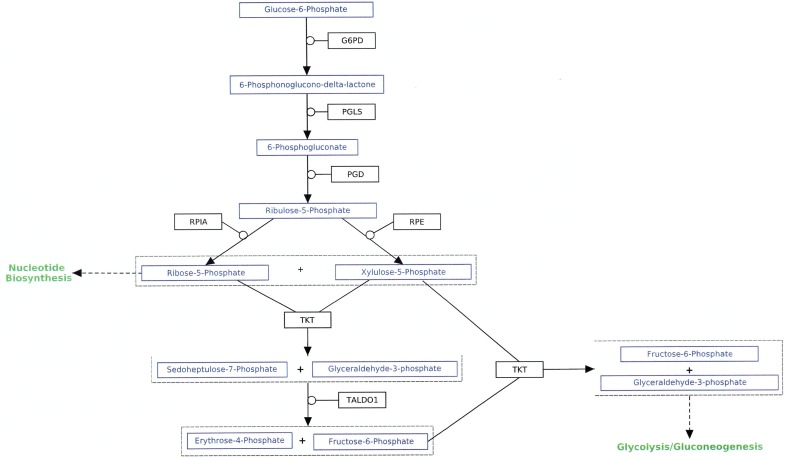
Pentose phosphate pathway (PPP) Taken from http://www.wikipathways.org/index.php/Pathway:WP134.

There are several reviews that suggest that a key role of aerobic glycolysis in cancer cells is to increase the flux of glucose metabolites through the PPP, thus increasing the production of the anti-oxidant NADPH as well as ribose-5-phosphate, a key precursor of nucleosides and nucleotides [see for example, 146,147]. There are two points of entry from glycolysis to the PPP. The first is from glucose 6-phosphate, which is converted to 6-phosphoglucono-δ -lactone by glucose-6-phosphate dehydrogenase in the first step of the PPP. This upstream portion of the PPP is known as the oxidative phase of the PPP and is responsible for the synthesis of NADPH. As noted above, an important advantage with respect to cellular proliferation resulting from the shift of PKM1 to PKM2 in cancer has been hypothesized to result from the decreased rate of the conversion of PEP to pyruvate, thus allowing upstream metabolites to accumulate and be metabolized via the PPP. Since it appears that the bottle neck at ALDOB would prevent increased formation of glucose-6-phosphate, increased flux through the PPP would have to be via the downstream entry point branching from glyceraldehyde-3-phosphate, the non-oxidative phase of the PPP. The possible importance of the non-oxidative phase of the PPP in cancer was clearly indicated in a paper by Boros et al. [148], which reported that 85% of the *de novo* synthesis of ribose in cultured Mia pancreatic adenocarcinoma cells is derived from glucose, and that the synthesis of ribose proceeds primarily (85%) via the non-oxidative phase of the PPP.

There are nine genes involved in the PPP (Supplementary Figure S1). Data were available for eight of these genes, the one exception being *RPE*, the gene that codes for the enzyme ribulose-phosphate 3-epimerase that catalyzes the conversion of ribulose-5-phosphate to xylulose-5-phosphate. Seven of these eight genes were neither over- nor under-expressed by greater than a factor of 2. The one exception was transketolase-like 2 (*TKTL2*), one of the three genes coding for proteins that catalyze the reversible conversion of ribose-5-phosphate and xylulose-5-phosphate to glyceraldehyde-3-phosphate and sedoheptulose-7-phosphate. This gene was under-expressed by a factor of 2.40; however, there is considerable scatter within the nine patients, and the result is not statistically significant (p = 0.067). Moreover, all calls were absent. There is almost no information in the literature regarding *TKTL2* or its corresponding protein. Langbein et al.[149] reported that *TKTL2* was strongly under-expressed (>10-fold) in tissues of three out of five human colon carcinomas compared to adjacent normal tissue as well as in two of five lung adenocarcinomas. In a slightly later paper Zhao et al. [150] reported that murine BCR-ABL transformed hematopoietic cell lines sensitive to imatinib and cultured under hypoxic conditions demonstrated a highly significant decrease in *tktl2* expression level following treatment with shRNA specific for *hif-1*α. This suggests that at least murine *tktl2* may be under the transcriptional control of hif-1α.

On the other hand there is considerable evidence that the transketolase-like 1 protein (TKTL1), which has also been reported to catalyze the formation of ribose-5-phosphate from glyceraldehyde-3-phosphate along with the protein transketolase (TKT), is up-regulated in a number of cancers [151-154]. Furthermore, oxythiamine, which has been reported to inhibit the activity of TKTL1, has been shown to inhibit tumor growth *in vivo* [155,156]. This finding has also been reported for other thiamine analogues [157]. There are limited data, however, for CCRCC. Langbein et al. [158] reported increased expression of TKTL1 protein in 55 kidney cancer patients using immunohistochemistry. Although the type of cancer was not specified, given that 70-80% of all kidney cancer is CCRCC, it is virtually certain that most of these cancers were indeed CCRCC. The average change in *TKTL1* gene expression level in the data set analyzed herein was an increase of only 1.15-fold that was clearly not statistically significant (p = 0.72). It is important to note, however, that all 18 calls were absent. Therefore, this result may not be particularly meaningful, as was the case with *TKTL2*. There is evidence that suggests that the role of TKTL1 may not be well understood. Mayer et al. [159] used real-time PCR to investigate the presence of the *TKTL1* gene in six different malignant cell lines and failed to find any evidence for the expression of this gene. In addition, they repeated the immunohistochemical studies using the same antibody used by the Langbein group [158] and reported staining of multiple unspecific bands in Western blots. These authors concluded that: “The data presented in this study raise reasonable doubts about the concept of the pathophysiological relevance of a transketolase isoenzyme TKTL-1 for energy metabolism, growth and progression of malignant tumors.” A later paper compared a computer model of the spatial structure of TKTL1 with TKT and concluded that it is unlikely that TKTL1 would be capable of catalyzing the transketolase reaction [160]. Jones and Schulze [161] have recently reviewed the evidence pro and con for the importance of TKTL1 in particular and the PPP in general in cancer. They conclude that: “Although the data support the role of the PPP in at least some types of cancer, the results also underline the importance of robust validation of potential cancer metabolism targets.” The data presented herein cannot resolve these possible issues.

In conclusion the data analyzed for CCRCC do not suggest any up-regulation of any part of the PPP, at least at the gene expression level. The only gene in this pathway with an average change greater than 2 was *TKTL2*, which was under-expressed. However, this change was not statistically significant, and its under-expression would seem to be of limited importance given that two other genes code for enzymes that catalyze the same reaction. The lack of any obvious increases in expression levels for any gene involved in the PPP is consistent with the conclusion that any proliferative advantage provided by aerobic glycolysis in CCRCC is not a consequence of increased synthesis of key biomolecules. However, the results do not provide proof of this conclusion. Unlike the situation with gluconeogenesis and the serine/glycine pathways, there is no evidence for the under-expression of key genes involved in the PPP. Therefore, it is certainly possible that increased levels of glyceraldehyde-3-phosphate produced by increased levels of PEP, as a consequence of the low reactivity of PKM2, would lead to increased flux through the non-oxidative arm of the PPP.

### Metabolism of Pyruvate

Pyruvate, formed by the action of PK on PEP, can undergo two principal routes of metabolism. The first is its decarboxylation to acetyl-CoA by the enzyme pyruvate dehydrogenase (PDH), while the second is its reduction to lactate by the enzyme lactate dehydrogenase (LDH). It is the shift in the partitioning of these two paths that led Warburg to conclude that the metabolism of glucose in cancer cells was radically different from its metabolism in normal cells, since cancer cells produced consistently high amounts of lactate even in the presence of oxygen [1]. In most adult somatic cells under normoxic conditions, pyruvate is transported to the mitochondria where it is metabolized to acetyl-CoA, which serves as a substrate for the TCA cycle. In such cells pyruvate is converted to lactate only when oxygen tension is low leading to an inhibition of oxidative phosphorylation. This section will cover the genes involved in the metabolism of pyruvate, including the TCA cycle. The results are provided in Table 4.

**Table 4:** Gene expression values relevant to pyruvate metabolism and the TCA cycle

Gene	FC Pat. 2	FC Pat. 3	FC Pat. 4	FC Pat. 5	FC Pat. 6	FC Pat. 9	FC Pat. 10	FC Pat. 11	FC Pat. 12	Average FC	p-value (from TDSIT)
**Over-expressed**											
*PDK1*	16.23	17.82	3.90	5.37	6.22	9.76	6.17	9.34	15.34	10.02	2.07E-06
*SLC16A3*	7.83	6.55	4.92	3.74	9.35	8.46	2.55	10.06	10.35	7.09	2.99E-06
*SLC16A1*	4.19	5.10	2.99	2.11	2.10	1.04	5.24	5.73	3.50	3.56	2.68E-04
*LDHA*	2.99	2.84	3.21	2.72	2.99	2.61	3.23	2.35	4.13	3.01	3.34E-08
**Under-expressed**											
*SUCLG1*	−4.57	−5.25	−4.71	−5.26	−5.22	−4.41	−2.62	−5.85	−6.42	−4.80	7.88E-08
*PDHB*	−1.76	−3.69	−1.59	−2.49	−3.63	−2.16	−2.03	−2.89	−4.51	−2.59	4.65E-05
*OGDH*	−2.14	−1.43	−1.44	−3.24	−1.77	−3.23	−5.35	−1.68	−3.64	−2.40	5.03E-04
*PDHA1*	−1.53	−2.65	−4.50	−1.35	−3.19	−2.18	−2.37	−2.85	−2.09	−2.38	1.04E-04
*SUCLG2*	−1.66	−2.84	−2.13	−1.72	−3.00	−2.92	−1.92	−1.98	−3.14	−2.30	9.97E-06
*DLST*	1.21	−2.03	−1.49	−2.51	−2.06	−4.19	−1.63	−2.62	−3.11	−2.08	1.60E-03
*FH*	−1.65	−3.89	−1.62	−2.13	−2.21	−2.60	−1.94	−1.32	−1.77	−2.03	1.48E-04
**Unchanged**											
*PDK4*	2.18	1.31	1.96	1.37	−1.71	1.98	2.11	1.67	−1.02	1.46	3.01E-02
*LDHB*	−2.27	−1.35	−1.84	−2.05	−3.05	−1.92	−1.74	−1.98	−1.18	−1.87	1.43E-04
*IDH2*	1.29	−1.86	−2.65	−1.98	−2.21	−3.29	−2.49	−1.44	−1.38	−1.86	2.51E-03
*PDK2*	−1.33	−1.57	−1.97	−1.56	−1.29	−1.79	−1.62	−1.75	−3.31	−1.73	3.56E-04
*ACO2*	−1.23	−2.03	−2.28	−1.66	−1.24	−3.15	−1.45	−1.47	−1.78	−1.73	6.57E-04
*SDHD*	−2.31	−1.11	−1.27	−1.21	−1.71	−1.82	−1.37	−1.80	−3.42	−1.67	2.49E-03
*DLAT*	1.05	−2.46	−1.66	2.73	−1.65	−2.07	−1.83	−1.67	−3.46	−1.55	7.51E-02
*SDHB*	−1.48	−1.99	−1.49	−1.23	−1.46	−1.78	−1.02	−2.09	−1.58	−1.53	4.53E-04
*SDHC*	1.72	−1.41	−1.78	−2.08	−1.03	−2.39	−1.74	−1.60	−2.05	−1.52	2.08E-02
*IDH3B*	−1.51	−1.20	−1.98	−1.23	−4.85	−1.23	−1.13	1.05	−1.33	−1.50	3.74E-02
*DLD*	−1.24	−1.16	−1.94	−1.03	−1.64	−2.18	−1.23	−1.46	−1.85	−1.48	1.93E-03
*SUCLA2*	−1.26	1.19	−1.31	1.09	−1.37	−1.32	−1.23	−2.23	−4.15	−1.44	5.43E-02
*MDH2*	−1.05	−1.07	−1.30	−1.48	−1.40	−1.52	−2.07	−1.49	−1.15	−1.36	2.32E-03
*SDHA*	1.01	1.14	−1.87	−1.88	1.10	−1.71	1.00	−1.66	−1.68	−1.33	3.25E-02
*IDH3G*	−1.21	−1.17	−1.28	−1.20	1.10	−1.47	−2.07	−1.34	−1.24	−1.29	8.11E-03
*CS*	−1.46	1.12	−1.52	−1.26	−1.34	−1.06	1.04	−1.02	1.22	−1.12	1.54E-01
*PDHX*	1.63	−1.02	−1.21	−1.12	−1.60	−1.23	−1.11	−1.27	−1.20	−1.12	2.17E-01
*IDH3A*	1.89	−1.09	−1.81	−1.42	1.28	1.04	−1.44	−1.42	1.56	−1.04	7.70E-01
*PDK3*	1.29	−1.36	1.11	−1.08	−1.52	1.47	−1.02	−1.22	1.10	−1.02	8.21E-01

#### Lactate dehydrogenase (LDH) and lactate transporter (MCT)

There are three separate genes, *LDHA/*C, that code for three monomeric forms of the lactate dehydrogenase enzymes LDHA-C. The active enzymes are all tetramers, and a number of specific isoenzymes have been described. Five isoenzymes consisting of combinations of LDHA (also known as LDHM (muscle)) and LDHB (also known as LDHH (heart)) have been described. These are LDH-1 (BBBB), LDH-2 (BBBA), LDH-3 (BBAA), LDH-4 (BAAA), and LDH-5 (AAAA) [162]. LDHC4 is specific to testis [19]. The LDHA protein chain is more active with respect to conversion of pyruvate to lactate in comparison to the B and C chains [163]. As anticipated, therefore, it is the *LDHA* gene that is found to be over-expressed in cancer. In addition, *LDHA* is under transcriptional control of HIF-1 [19]. There are a number of results indicating that levels of the LDH-5 protein (AAAA) are of prognostic value for a number of different types of cancer including melanoma [164] and squamous cell head and neck cancer [165]. Our analysis confirmed these findings, with *LDHA* being over-expressed by a factor of 3 (p = 3.34E-08) in the nine renal cancer patients. Proteomic studies comparing levels of LDHA in RCC tissue and normal adjacent tissue also observed an increase in this protein in RCC by a factor of 3.3-21.1, consistent with the gene expression results [66,77]. On the other hand, *LDHB* was under-expressed. The average of the nine patients was slightly below 2 (FC = −1.87); however, the result was statistically significant (p = 1.43E-04). Although *LDHC* was also somewhat over-expressed (FC = 2.24), all calls were absent; therefore, this over-expression is of limited importance.

Another piece of evidence establishing that CCRCC tumor tissue metabolizes glucose primarily to lactate as opposed to acetyl-CoA involves the lactate transporter proteins MCT1 and MCT4, which have been reported to remove lactate from cancer cells characterized by the aerobic glycolytic phenotype. These proteins are members of the monocarboxylate transport family. They are proton-linked 12-span transmembrane proteins and are not specific for lactate. Although 14 members of this family are known, research on these proteins has been essentially focused on MCT1-4. MCT2 and MCT3 primarily function to import lactate into cells, whereas MCT4 is effective in the transport of lactate out of cells. There are data that suggest that MCT1 can transfer lactate in either direction [166]. Pinheiro et al. [167] examined the levels of MCT1, MCT2, and MCT4 in about 120 tumor samples (breast carcinoma, colon adenocarcinoma, non-small lung cancer, and ovarian adenocarcinoma) using immunohistochemistry. Protein levels of MCT1 and MCT4 were significantly increased in tumor tissue compared to normal adjacent tissue in both breast and lung cancer tissues (p = 0.001). These authors note that only in the case of MCT1 was there an increase localized to the plasma membrane, which they indicate is a requirement for lactate transport. A somewhat earlier paper concluded that MCT4 is adapted to the transport of lactate from glycolytic cells, although this conclusion is based on the use of normal somatic cells with high rates of glycolysis as opposed to tumor cells [168]. The *SLC16A* gene family codes for the MCT proteins. The gene that codes for MCT1, *SLC16A1*, was found to be over-expressed by more than a factor of 3 (p = 2.68E-04) in the nine patients investigated, while the gene that codes for MCT4, *SLC16A3*, by a factor of 7 (p = 2.99E-06). There is recent independent confirmation with respect to an increase of MCT4 in CCRCC on both the gene and the protein level. Gerlinger et al. [169] reported that *SLC16A3* was the most highly and consistently over-expressed gene comparing results from 59 CCRCC samples to 11 normal kidney tissue samples. On the other hand they report no increase in the expression of *SLC16A1*. More recently Fisel et al. [170] reported a highly significant increase in MCT4 protein levels determined by immunohistochemistry in two different large patient cohorts in CCRCC tissue compared to normal adjacent tissue (p<0.0001). In addition, there was a significant correlation between MCT4 up-regulation and cancer-related death. Similarly, there was a highly significant increase in *SLC16A3* expression in a third cohort (p<0.00001). These results taken together with our analysis clearly support that pyruvate is primarily converted to lactate in CCRCC.

#### Pyruvate dehydrogenase (PDH) and pyruvate dehydrogenase kinase (PDK)

In cellular respiration, pyruvate is transported to the mitochondria where it is converted into acetyl-CoA, which then is utilized by the TCA cycle to complete the metabolism of glucose to CO_2_ and water, accompanied by the synthesis of 36 molecules of ATP for each glucose molecule. The protein responsible for this conversion is pyruvate dehydrogenase (PDH), which is a complex of three major subunits designated as E1-3. The E1 subunit is composed of two α subunits and one β subunit. There are five distinct genes that code for these subunits. *PDHA1* encodes the protein PDHE1-A type I, which constitutes the α1 subunit of E1, the active site of the PDH protein. *PDHA2* encodes the protein PDHE1-A type II, which also constitutes the α1 subunit of E1 but is specific for testis. *PDHB* encodes the protein PDHE1-B, which constitutes the β subunit of E1. *DLAT* encodes the protein PDHE2, which constitutes the E2 subunit, and lastly *PDHX* encodes PDHX, the E3 subunit. It is well established that in tumors PDH is inhibited by phosphorylation of PDHE1A by pyruvate dehydrogenase kinase (PDK). Four different isoenzymes of PDK have been described encoded by the genes *PDK1/4*. Our results have shown that *PDK1* was over-expressed in tumor tissue by a factor of 10 (p = 2.07E-06). This is consistent with the fact that *PDK1* has been reported to be under the transcriptional control of HIF-1 [171]. None of the other *PDK* genes were changed by a factor of 2, with *PDK2* being slightly under-expressed and *PDK4* being slightly over-expressed (Table 4). Although the inactivation of the PDH proteins is a posttranscriptional modification, two of the five genes coding for the five PDH isozymes were also under-expressed by at least a factor of two; namely, *PDHB* and *PDHA1* (FC = −2.59, p = 4.65E-05 and FC = −2.38, p = 1.04E-04). Given that *PDHA1* codes for the active site of the PDH protein, its down regulation is of some interest. Two of the three remaining three *PDH* genes, *DLAT* and *PDHX*, were also under-expressed but by less than a factor of 2, while *PDHA2* was characterized by only absent calls.

#### TCA Cycle

**Figure 4:**
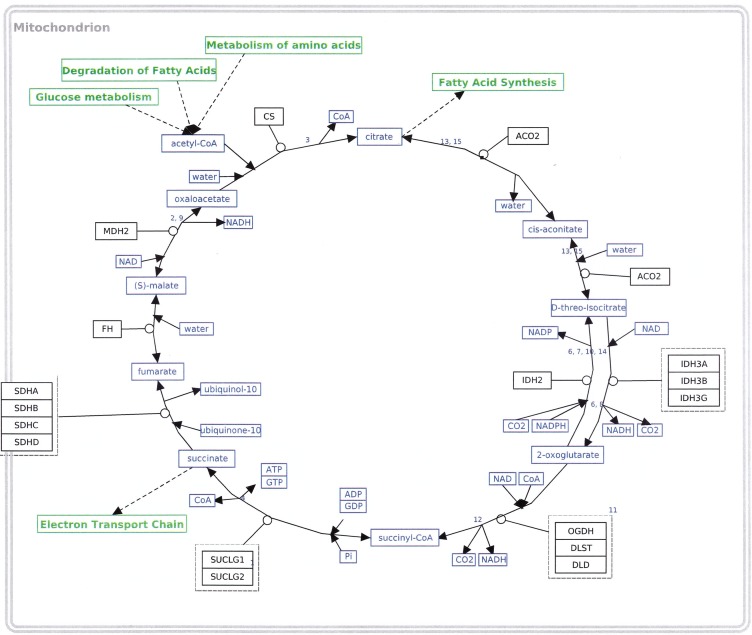
Tricarboxylic acid (TCA) cycle Taken from http://www.wikipathways.org/

A total of 18 genes are involved in the TCA cycle (Figure 4), and data for all 18 were available. As shown in Table 4, every gene in the TCA cycle without exception was under-expressed. Five genes were under-expressed by at least a factor of 2. The first two of these five under-expressed genes, *OGDH* and *DLST*, code for two of the three protein components of the 2-oxoglutarate dehydrogenase complex. This complex catalyzes the conversion of 2-oxoglutarate to succinyl-CoA in the presence of CoA. These two genes had FCs of −2.40 (p = 5.03E-04) and −2.08 (p = 1.60E-03), respectively. Two of the three genes that code for the α and β subunits of the heterodimeric protein succinate coenzyme A ligase (SUCL) were also significantly under-expressed. SUCL catalyzes the reversible conversion of succinyl-CoA to succinate and is encoded for by the two genes, *SUCLG1/2*, which exhibited FCs of −4.80 (p = 7.88E-08) and −2.30 (p = 9.97E-06), respectively. Lastly, *FH*, the gene that codes for the enzyme fumarate hydratase was also under-expressed by greater than a factor of 2 (FC = −2.03, p = 1.48E-04). This well-known protein catalyzes the reversible conversion of fumarate to (S)-malate.

Warburg's early experiments with murine ascites cancer cells, demonstrated not only a highly significant increase in the production of lactate but also a highly decreased utilization of oxygen [1]. He proposed that this decrease was a consequence of irreversible damage to the cells' respiration, in other words loss of function of the TCA cycle or oxidative phosphorylation. Many exceptions violating this conclusion have been found in the following decades, and the subject has been recently reviewed [172,173]. Moreno-Sanchez et al. [172] point out that some types of tumor cells, such as glioma C6 cells and LoVo colon adenocarcinoma cells, produce ATP primarily by glycolysis, whereas other types, such as bone sarcoma cells and lung carcinoma cells, produce ATP primarily by oxidative phosphorylation. Neither of these reviews provides any information relevant for CCRCC tissue or cells. In addition, as will be discussed below, the TCA cycle can function quite well in cells where pyruvate-derived acetyl-CoA is prevented from entering the mitochondria by using glutamine as a substrate. This, as well as the results obtained for *SUCLG1*, will be discussed in more detail below.

### Lipid Synthesis, Anaplerosis, Cataplerosis, and Glutaminolysis (Table 5)

**Table 5:** Gene expression values relevant to lipid synthesis, anaplerosis, cataplerosis, and glutaminolysis

Gene	FC Pat. 2	FC Pat. 3	FC Pat. 4	FC Pat. 5	FC Pat. 6	FC Pat. 9	FC Pat. 10	FC Pat. 11	FC Pat. 12	Average FC	p-value (from TDSIT)
**Over-expressed**											
*ACLY*	2.15	3.23	1.62	3.07	2.44	1.97	3.13	1.87	3.54	2.56	1.05E-05
**Under-expressed**											
*ACO1*	−2.98	−2.83	−2.60	−2.59	−2.99	−5.76	−1.53	−2.13	−2.88	−2.76	2.38E-05
*GLS*	−2.81	−1.90	−3.53	−2.49	−4.43	−2.45	−1.54	−1.98	−3.71	−2.62	3.28E-05
*GLS2 (1)*	−1.86	−3.98	−1.40	−7.23	−4.00	−2.63	−2.27	1.28	−3.18	−2.54	2.65E-03
*ACACB*	−2.29	−2.88	−2.28	−3.10	−4.20	1.10	−1.89	−3.43	−1.79	−2.32	5.08E-04
*IDH1*	−2.69	−2.43	−2.21	−2.95	−3.58	−1.68	−1.22	−1.67	−2.30	−2.20	9.61E-05
*GOT1*	−1.09	−1.72	−3.16	−1.55	−3.59	−2.92	−1.27	−1.86	−2.75	−2.04	1.02E-03
**Unchanged**											
*ME2*	1.83	1.45	1.40	1.62	1.53	1.09	1.18	1.25	2.20	1.47	7.54E-04
*GOT2*	−1.25	−1.18	−2.32	−1.66	−2.46	−2.28	−2.66	−1.86	−1.92	−1.89	1.76E-04
*GLUD1*	−1.57	−1.45	−1.02	−1.39	1.57	−4.25	−2.15	1.07	−2.89	−1.55	5.61E-02
*FASN*	−1.22	−1.57	−1.09	−1.61	1.32	−1.28	−2.05	1.79	1.11	−1.14	3.48E-01
*ME1*	−1.40	−1.01	1.94	1.25	−2.32	−1.54	−1.34	2.68	−1.21	−1.03	9.00E-01

(1) All calls were absent

Although the proteins involved in the TCA cycle are located exclusively in the mitochondria, certain TCA metabolites can be exported to the cytosol in a process known as cataplerosis. These metabolites must be replaced for the TCA cycle to properly function, and this process is known as anaplerosis [174]. A key TCA metabolite that undergoes cataplerosis is citrate. This metabolite is required for lipid synthesis, and the first step of this process, which takes place in the cytosol, is its conversion to acetyl-CoA and oxaloacetate catalyzed by the protein ATP citrate synthase (ACL). This protein is coded for by the gene *ACLY*.

There is considerable evidence that lipid synthesis is increased in rapidly proliferating cells that exhibit the aerobic glycolytic phenotype [175], and a number of different types of cancers, including lung, prostate, bladder, breast, liver, stomach, and colon cancer, exhibit over-expression of *ACLY* [176]. Moreover, inhibition of the ACL protein has been shown to inhibit cancer cell proliferation both *in vitro* and *in vivo* [175,177,178]. The gene expression data from the nine CCRCC patients included in our analysis were consistent with these results, with a 2.56-fold increase in *ACLY* expression (p = 1.05E-05). On the other hand, the genes that code for the proteins that catalyze the following two steps of lipid synthesis were not over-expressed. With respect to the first of these steps, acetyl-CoA carboxylase beta (*ACACB*), which codes for acetyl-CoA carboxylase 2 (ACC2), one of the two isoforms of the enzyme that converts citrate-derived acetyl-CoA to malonyl-CoA, was actually under-expressed (FC = −2.26, p =5.60E-04). The expression level of acetyl-CoA carboxylase alpha (*ACACA*), which codes for the other isoform of the enzyme acetyl-CoA carboxylase (ACC1), could not be determined, since all 18 calls were absent. Expression of the gene fatty acid synthase (*FASN*), which codes for the protein FAS, was essentially unchanged. This enzyme catalyzes the following step of fatty acid synthesis; namely, the synthesis of long chain fatty acids from acetyl-CoA and malonyl-CoA in the presence of NADPH. It is possible that the sample of CCRC tumors used in this study was too small and not sufficiently diversified with respect to stage to detect an increase in *FASN* expression. Horiguchi et al. [179] reported finding positive FAS protein staining in 18% of 120 renal tumors. This expression was associated with advanced pathological T stage, regional lymph node metastasis, and distant metastasis. In a more recent study, Hakimi et al. [180] observed increased *FASN* expression to be associated with poor survival rates in renal cancer patients. Nevertheless, these results may call into question that over-expression of *ACLY* observed in the nine CCRCC patients examined in this study is indicative of increased lipogenesis. The expression level of *SLC25A1*, which codes for the tricarboxylate transport protein, could have provided evidence as to whether or not citrate is indeed being transported into the cytosol. Unfortunately, that gene was not included in the data set analyzed.

It should be noted that there is some evidence of another function of ACL that could play a role in shifting cellular metabolism to anaerobic glycolysis. Wellen et al. [181] reported that inhibition of this enzyme led to a global decrease of histone acetylation. Of particular importance was that levels of four specific proteins involved in aerobic glycolysis were also decreased; namely, GLUT4, HK2, PFK-1, and LDHA. Therefore, it is possible that the over-expression of *ACLY* that occurs in CCRCC leads to an increase in histone acetylation that increases transcription of these four genes, thus contributing to the aerobic glycolytic phenotype.

If over-expression of *ACLY* denotes increased levels of ACL protein being used to degrade citrate to acetyl-CoA, then the citrate that is exported from the mitochondria (cataplerosis) must be replaced (anaplerosis). Two small molecules have been identified as being key contributors to anaplerosis. Although glutamine is most frequently cited as being an anaplerotic substrate, pyruvate can fulfil this role as well. Cheng et al. [182] found that when *GLS*, the gene that codes for the enzyme required for the first step of analplerotic utilization of glutamine, was suppressed using shRNA in either LN229 or SF188 glioblastoma cell lines, net glutamine utilization and cell proliferation were both reduced, yet the cells remained completely viable. These authors reported a source of glucose-derived anaplerosis; namely, the conversion of pyruvate to oxaloacetate in the mitochondria by pyruvate carboxylase (PC). The importance of pyruvate as an anaplerotic substrate has also been reported in other cell lines [183]. This conversion of pyruvate to oxaloacetate was discussed above, in that it is the first step of gluconeogenesis, and it was also noted that the *PC* gene was very highly under-expressed in the nine CCRCC patients (Table 3). Therefore, this reaction is clearly not serving as a source of oxaloacetate, which could then be converted to citrate thus replacing the citrate exported for the first step of the fatty acid synthetic pathway.

As noted above, glutamine is generally regarded as being the major anaplerotic substrate [183-186]. Glutamine is transported to the mitochondria where it is converted to glutamate. Two genes are involved in this transformation, *GLS* (also known as *GLS1*) and *GLS2*. *GLS* codes for 2 proteins, KGA (also known as kidney type (K-type) glutaminase) and GAC (also known as glutaminase C), that are splice varieties. *GLS2* also codes for 2 proteins, LGA and GAB, both of which are often referred to as liver type enzymes. In this case the two proteins are transcribed from the *GLS2* gene but under the control of different promoters. Interestingly, the *GLS* and *GLS2* genes as well as their corresponding proteins appear to be associated with virtually diametrically opposed cellular phenotypes. *GLS*-derived protein up-regulation is associated with increased rates of cellular proliferation, whereas *GLS2* prevalence appears to be associated with resting, non-proliferative, or quiescent cell states. It has been reported, for example, that over-expression of the human *GLS2* gene in the T98 glioblastoma cell line correlated with a reversion of the transformed phenotype [187]. On the other hand, there is abundant *in vitro* and *in vivo* evidence that inhibition of the proteins coded for by *GLS* inhibits cellular proliferation [183,187,188].

It might be assumed, therefore, that *GLS* would be over-expressed in CCRCC, whereas *GLS2* would be under-expressed or unchanged. This, however, was not the case. *GLS* was actually significantly under-expressed (FC = −2.62, p = 3.28E-05) in the nine CCRCC patients that provided the data for this study. *GLS2* was also significantly under-expressed, although to a very slightly lesser extent (FC = −2.54, p = 2.52E-03). It should be noted, however, that all calls for *GLS2* were absent. The fact that *GLS* is under-expressed would seem to suggest that glutamine does not act as an anaplerotic substrate in CCRCC. Further evidence supporting this conclusion is that there was essentially no change in expression level of *GLUD1* (FC = −1.33), which codes for the protein glutamate dehydrogenase (GLD1, also known as GLDH). This protein catalyzes the conversion of glutamate to α-ketoglutarate, thus completing the anaplerotic process. There is very little literature information with respect to the role of either *GLUD1* or GLD1 in cancer. It has been reported, however, that the activity of GLD1 in peripheral blood mononuclear cells is increased in individuals with untreated B-chronic lymphocytic leukemia as compared to healthy controls [189].

Further information regarding the role of glutamine might have been provided from the gene expression level of the gene coding for the protein responsible for the transport of glutamine from the cytosol to the mitochondria. This, however, turns out not to be the case. First of all there are numerous transporters that have been identified for glutamine, but none of them are specific for glutamine. Secondly, the protein that transports glutamine into the mitochondria has not yet been identified [190]. One transporter that appears to play a key role in tumor cells is ASCT2, coded for by the gene *SLC1A5* [190-192]. However, it is highly likely that the role that ASCT2 is playing involves the role of glutamine with respect to mTOR signaling, and this will be discussed briefly below.

Previously, this analysis has taken the position that if a gene is under-expressed by greater than a factor of two and the change is statistically significant, the process catalyzed by the enzyme coded for by such a gene is not playing a key role in the tumor. However, there are several reasons why glutamine may still be a precursor of citrate in CCRCC despite the under-expression of *GLS* by a factor of 2.62. One point is that protein activity does not necessarily correlate with gene expression level. For example, Erickson and Cerione [193] reported that there is a marked increase of the glutaminase enzyme GAC as a consequence of phosphorylation of the enzyme. Secondly, Wise et al. [194] recently published that glutamine uptake into mitochondria is stimulated by the oncoprotein c-Myc in SF188 glioma cells, while Gao et al. [195] reported that the c-Myc enhanced production of mitochondrial glutaminase was not a consequence of enhanced *GLS* expression but rather a result of the suppression of miR-23a/b, which apparently can decrease the translation of *GLS* mRNA. However, perhaps the most important point is that the normal kidney consumes very large amounts of glutamine. Glutamine enters the kidney mitochondria where it is transformed through the process of glutaminolysis, which will be discussed below, and eventually serves as a major source for total glycogen [196,197]. Moreover, it is required to maintain acid-base balance via the production of ammonia [198]. Given that glutaminolysis is not contributing to glycogen synthesis in CCRCC, since it has already been demonstrated that those genes that code for unique enzymes involved in gluconeogenesis are strongly under-expressed, a relatively small decrease in expression value for *GLS*, may still allow an ample supply of glutamate to be formed allowing glutamine to serve as an anaplerotic substrate.

Although the canonical pathway for the replacement of citrate by glutamine involves incorporation of glutamate in the TCA via the GLD1 conversion of glutamate to 2-oxoglutarate followed by its conversion to isocitrate and then citrate in the oxidative direction, there exists another pathway by which glutamine can be converted to isocitrate. Moreover, there is evidence that this pathway is of particular importance in tumors that are either hypoxic or pseudohypoxic. This pathway, known as reductive carboxylation, involves conversion of glutamine to citrate via the reductive TCA cycle. One of the key steps in this reaction is the IDH catalyzed conversion of 2-oxoglutarate to isocitrate. In principle, this reaction can occur in the mitochondria, catalyzed by IDH2 and/or IDH3, or in the cytosol, in which case it would be catalyzed by IDH1. Metallo et al. [199] used labelling studies to demonstrate that the citrate-derived acetyl-CoA used in fatty acid synthesis was derived from the reductive carboxylation of 2-oxoglutarate in a large number of cancer cell lines. In addition, they showed that knockdown of mRNA from *IDH1* was effective in reducing this reductive carboxylation and impaired cellular proliferation in these cell lines. On the other hand, knockdown of mRNA derived from *IDH2* did not affect reductive flux in A549, MDA-MB-231, and HCT116 cells. These results strongly suggest that reductive formation of citrate occurs in the cytosol and not the mitochondria. Of particular interest is the fact that there was a significant increase in reductive carboxylation when cells were cultured under hypoxia. The role of pseudohypoxia with respect to reductive carboxylation was also demonstrated by Gameiro et al. [200]. These authors reported that labelling studies confirmed the formation of citrate in UMRC2 cells, a *VHL*-deficient human renal carcinoma cell line. Such cells would, by definition, exhibit pseudohypoxia. Reintroduction of wild-type pVHL suppressed the contribution of reductive carboxylation. These authors do not provide any information as to which isoenzyme of IDH is active in this system. Another recent study investigated the role of reductive carboxylation in two different melanoma cells lines [201]. This study reported that the reductive pathway did not play a role in either WM35 or LU1205 cells under normoxic conditions, but did play an important role under hypoxic conditions. These authors reported that both IDH2 and IDH1 were responsible for the reductive conversion of glutamine to citrate. A fourth report indicated that reductive carboxylation was catalyzed specifically by IDH2 in SF188 glioblastoma cells cultured under hypoxic conditions [202]. All of these studies may be correct, in that different cell lines were used. However, it should be noted that a recent paper by Moreno-Sanchez et al. [203] suggests that the change in free energy of the conversion of isocitrate to 2-oxoglutarate is too large to allow it proceed in the reverse direction in the mitochondria. Conversion in the cytosol, however, would be much more likely.

If 2-oxolgutarate is serving as the precursor for isocitrate in the cytoplasm, then it must be able to cross the mitochondrial membrane. This can be accomplished by the mitochondrial 2-oxoglutarate/malate carrier protein (OGCP) coded for by the gene *SLC25A11* [204]. Although the canonical pathway for the conversion of glutamate to 2-oxoglutarate is via its oxidation by *GLUD1*, as already mentioned, there is another route. This route involves the transaminase aspartate aminotransferase (AAT, also known as AST, ASAT, GOT), which involves the reaction of glutamate with oxaloacetate to produce 2-oxoglutarate and aspartate. The soluble form of this enzyme is coded for by the gene *GOT1*, while the mitochondrial form is coded for by the gene *GOT2*. It has been recently reported that AAT catalyzes the conversion of glutamate to 2-oxoglutarate in human pancreatic ductal adenocarcinoma [205,206]. The authors describe this route as a novel pathway. There is some indication, however, that this pathway is operative in the HuH13 human hepatoma cell line [207], as well as in transformed NIH3T3 mouse fibroblasts expressing an oncogenic K-Ras protein [208].

Gene expression values for the cytosolic reductive carboxylation pathway were calculated for the nine patients analyzed. The key gene with respect to conversion of 2-oxoglutarate to isocitrate in the cytoplasm is *IDH1*. This gene was clearly under-expressed in tumor tissue with a FC of −2.20 (p = 9.61E-05). In addition, *ACO1*, the gene that codes for the cytosolic form of aconitase, the enzyme that catalyzes the conversion of isocitrate to citrate, was also clearly under-expressed (FC = −2.76, p = 2.38E-05). Therefore, it seems clear that reductive carboxylation of 2-oxoglutrate does not occur in CCRCC tumor tissue from these nine patients. Further confirmatory evidence could have been provided by the expression results for *SLC25A11*, the gene that codes for the transport protein that allows 2-oxoglutarate to move from the mitochondria to the cytoplasm in exchange for malate. Unfortunately, data for this gene was not included in the data set. No conclusions can be drawn with respect to any role of mitochondrial reductive carboxylation of 2-oxoglutarate. This is because it is impossible to determine if any change in gene expression level involves flow through the TCA cycle in the oxidative or reductive direction. In any event, all of the genes involved were slightly under-expressed (*IDH2*, FC = −1.86, p = 2.51E-03; *IDH3A*, FC = −1.04, p = 7.70E-01; *IDH3B*, FC = −1.50, p = 3.74E-02; *IDH3G*, FC = −1.29, p = 8.11E-03; *ACO2*, FC = −1.73, p = 6.57E-03).

There is also no evidence to suggest that the non-canonical conversion of glutamate to 2-oxoglutarate catalyzed by either the cytosolic or mitochondrial form of AAT occurs in the tumor tissue of these nine CCRCC patients. The gene *GOT1* that codes for the cytosolic form of AAT is under-expressed by a factor of 2.04 (p = 1.02E-03), while *GOT2*, which codes for the mitochondrial form of AAT, is under-expressed by close to a factor of 2 (FC = −1.89, p = 1.76E-04). A recent proteomic study reported down-regulation of GOT2 protein in renal cancer tumor tissue when compared to matched normal tissue [209].

To summarize, *ACLY* was over-expressed in tumor tissue suggesting that citrate is being exported from the mitochondria to the cytosol, where it is converted to acetyl-CoA, which serves as a starting point for lipid synthesis. The fact that the genes that code for the proteins that catalyze the following two steps of lipid synthesis were not over-expressed casts some doubt on this straightforward explanation for the increase in *ACLY* expression. This doubt is reinforced by the fact that both *PC* and *GLS*, which code for proteins allowing pyruvate and glutamate, respectively, to serve as anaplerotic substrates, were also under-expressed in tumor tissue. However, it is difficult to draw a definitive conclusion. As pointed out above, there is evidence that the protein FAS is not up-regulated in early stage renal cancer, but that it is up-regulated in later stage disease. Given that all nine of the patients analyzed herein had either stage 1 or stage 2 disease, the fact that *FASN* expression was unchanged is consistent with the protein data. Secondly, the under-expression of *GLS* may be difficult to interpret in that the normal kidney clearly metabolizes large amounts of glutamine via the mitochondria. It does appear clear, however, that *GLS* or its corresponding protein would not be a viable target to treat CCRCC. By the same token, reductive carboxylation of 2-oxoglutarate and conversion of glutamate to 2-oxoglutarate by transamination would also not provide meaningful targets. *ACLY*, on the other hand, may be a viable target. Further research into the role that *ACLY* and its corresponding protein are playing in CCRCC could be of considerable importance.

It is important to point out that glutamine plays a large number of roles in both normal and tumor tissue – particularly tumor tissue. Several authors have stated that cancer cells are addicted to glutamine. One of the principal functions of glutamine in cancer is the glutaminolysis pathway [185,198]. The first two steps of glutaminolysis, conversion of glutamine to 2-oxoglutarate via glutamate, have already been described in considerable detail above in the context of glutamine as an anaplerotic substrate to replace citrate. However, glutaminolysis can serve as a major source of energy via its metabolism through the TCA cycle, particularly under conditions where the availability of pyruvate-derived acetyl-CoA is reduced stemming from glucose deprivation, hypoxia, or pseudohypoxia. In addition, glutamine serves as a major source of NADPH, thus maintaining redox balance. There is essentially no information that the present gene expression data can contribute to elucidating the role of most of the remaining steps of glutaminolysis in CCRCC. This is because the majority of these steps constitute the TCA cycle. As discussed above, all genes coding for TCA cycle proteins were under-expressed, but only five of them were under-expressed by more than a factor of 2. Four of these five genes were under-expressed by less than a factor of 2.5. The one exception is the gene *SUCLG1*, which was under-expressed by almost a factor of 5 (FC = −4.80, p = 7.88E-08). This gene codes for the α-subunit of the protein SUCL, which catalyzes the conversion of succinyl-CoA to succinate. The marked under-expression of *SUCLG1* would suggest that the TCA cycle is not functional in these nine CCRC patients. However, that conclusion is not consistent with the gene expression levels of the remaining 17 genes involved in the TCA cycle. On the other hand a recent publication that compared 30 matched tumor and normal tissue samples reported that the protein SUCLG1 was down-regulated by a factor of 13, which fully supports our gene expression analysis [209]. There is one pathway involved in glutaminolysis that is external to the TCA cycle. This pathway is the conversion of malate to pyruvate via malic enzyme, an NAD+ dependent enzyme that also produces NADPH. One recent study reported that a significant amount of pyruvate produced by malic enzyme is converted into lactate [210], while another study reported a relatively small amount of lactate from this route [211]. Although both studies utilized SF188 glioblastoma cells, it is difficult to compare the two results. There are two forms of malic enzyme, a cytosolic form, which is coded for by the gene *ME1*, and a mitochondrial form coded for by *ME2*. Our analysis indicated that the expression level of *ME1* is unchanged (FC = −1.03, p = 0.9). *ME2*, on the other hand is somewhat over-expressed (FC = 1.47, p = 7.54E-04) but by less than a factor of 2. As can be seen, it is rather difficult to draw any firm conclusions as to the role that glutaminolysis plays in CCRCC based on these data. It may play a role, but the gene expression result for *SUCLG1* casts some doubt on this. Perhaps there is an unknown protein that can catalyze the conversion of succinyl-CoA to succinate.

Glutamine plays many other roles, some of which are important in cancer. Two of them will be briefly mentioned, although they have no real relationship to aerobic glycolysis. The first is that glutamine is a key precursor of glutathione, necessary to detoxify ROS [212]. A second and highly interesting role of glutamine is that it has been shown to induce mTOR signaling via mTORC1 thus increasing cellular proliferation. This has been reported in HeLa cells [213], A549 lung cancer cells [214], and C8161 and 1205Lu melanoma cells [215]. Somewhat curiously, it appears that glutamine itself has no effect on mTORC1. Glutamine is taken up by the cell via the transport protein ASCT2, and then is exchanged for leucine by the transport protein LAT1 [191,213]. It is then leucine that induces mTOR signaling. As can be seen, glutamine is an extremely important molecule in cancer. Some effects are related to glucose metabolism, while others are completely unrelated. As indicated, however, the contributions of glutamine to the pathway of glucose metabolism are extremely difficult to define in CCRCC based on gene expression data. Other roles, although possibly important with respect to CCRCC, are beyond the scope of this analysis.

## CONCLUSIONS

The results outlined above are quite gratifying in that they are consistent with the expectation that nine out of ten subjects with CCRCC in the data set analyzed clearly exhibit a Warburg effect. Moreover, in general, the gene expression changes were in line with the copious literature dealing with aerobic glycolysis. The majority of the genes that code for glycolytic enzymes as well as for LDH were over-expressed, whereas the genes that code for the proteins involved in the dehydrogenation of pyruvate to yield acetyl-CoA were under-expressed. *PDK1*, the gene that codes for the enzyme that phosphorylates PDH thereby inhibiting pyruvate dehydrogenase, was also highly over-expressed. The genes that code for the proteins involved in the TCA cycle were either under-expressed or unchanged. These results point to an increased metabolism via glycolysis to pyruvate and lactate in the CCRCC tumor tissue investigated. However, there are also a few surprising results. The first is that *ENO2* is very highly over-expressed (FC = 33.79). This gene codes for the protein γ-enolase, which is known to be highly up-regulated in tumors of neuroendocrine cells. There are previous reports that γ-enolase is indeed up-regulated in renal cancers, although the reported increase is much more modest. Moreover, there is evidence suggesting that CCRCC is not of neuroendocrine cell origin. Although at this time there appears to be no good explanation of why *ENO2* is so highly over-expressed, it would appear that the *ENO2*/γ-enolase axis would constitute a promising target for anti-cancer drugs.

The second surprise involves the four *PFKFB1/4* genes. Our findings indicate that *PFKFB4* was significantly over-expressed (FC = 3.00), whereas *PFKFB2* was highly under-expressed (FC = −5.02). Most published results have reported that the *PFKFB* gene over-expressed in cancer is *PFKFB3*, although, as noted, a recent result was cited that indicated over-expression of *PFKFB4* in three different prostate cancer cell lines [58]. The CCRCC data presented herein indicate that *PFKFB3* is somewhat over-expressed but by less than a factor of 2. Our analysis suggests that the role of the four *PFKFB* genes in cancer may not be well understood. Nevertheless, drugs that might decrease the expression of *PFKFB4* or its corresponding protein or increase the expression of *PFKFB2* or its corresponding protein could be of interest with respect to CCRCC.

The most surprising result, however, relates to the *PKM* gene. Although this gene was indeed over-expressed in CCRCC tissue (FC = 3.19), since this gene codes for both PKM1 and PKM2, no conclusions can be drawn as to which of the two proteins might be up-regulated. On the other hand genes that code for the four proteins that are responsible for the alternate splicing leading to the production of PKM2 were not over-expressed. That could imply that either the protein expressed in normal tissue is already PKM2, as suggested by Bluemlein et al. [112], or that there is no shift from PKM1 to PKM2 in the tumor. As discussed above, however, either conclusion would be at considerable odds with extensive proteomic data. Nevertheless, what does appear to be a valid conclusion is that if there is a shift from PKM1 to PKM2, the lowered activity of PKM2 as compared to PKM1 does not provide CCRCC tumor cells with a proliferative advantage by forcing a buildup of metabolites that can be used to synthesize key biomolecules such as nucleotides and amino acids. This would require that the glycolysis pathway proceeds in the reverse direction to allow the relevant metabolites to be diverted into side pathways responsible for the synthesis of such biomolecules. The gene expression values derived from these nine CCRCC patients did not support this hypothesis. First of all, the unique genes involved in gluconeogenesis, the reverse of glycolysis, were all significantly under-expressed in CCRCC tissue compared to adjacent normal tissue (Table 3). Moreover, most of the genes in one of the pathways branching from glycolysis that has been implicated as a source of key biomolecules, the serine/glycine pathway, were also clearly under-expressed (Table 2). Genes involved in the second key pathway, the PPP, which has been proposed as a source of ribose-5-phosphate, a key precursor of nucleosides and nucleotides, were essentially unchanged. In addition, it would appear logical to assume that cancer cells would require energy in the form of ATP to proliferate. Given that PMK2 has reduced activity compared to PKM1 would suggest a decrease in available ATP. Taken together, these data would suggest that PKM2 would not be a profitable target with respect to CCRCC. This conclusion, however, may need to be modified by the fact that there are recent findings that PKM2 can be translocated to the nucleus, particularly the “inactive” dimeric form. The exact role of nuclear PKM2 is not known; however, evidence has been published that it can regulate HIF-1 transcriptional activity [216] and interact with and activate other transcription factors such as β-catenin, Oct-4, and Stat3, thereby contributing to cell survival and proliferation [217].

It would seem obvious that certain cancers adopt the aerobic glycolytic phenotype to provide the tumor with a competitive advantage. It does not appear, however, that the overall changes to the metabolism of glucose provides CCRCC with such an advantage through increased synthesis of key biomolecules required for increased proliferation. A key question, therefore, is how do these tumors proliferate? Part of the answer is that early stage renal tumors grow very slowly [218,219]. Organ et al. [219] reported that Fuhrman stage 1 tumors (<7 cm) from 169 patients grew at a median rate of 0.12 cm/year. Although four of the nine patients analyzed in this study had Fuhrman stage 2 cancer (patient 8 was not analyzed), we detected no differences in gene expression patterns between stage 1 and stage 2 patients. This point was also made in one of the two papers published by the investigators who deposited the data in the GEO [9]. A possible explanation for the competitive advantage imparted by aerobic glycolysis in CCRCC is that the increased production of lactic acid could be playing a key role. There is considerable support for this hypothesis. One of the earliest papers to focus on a possible competitive advantage provided to cancer cells by lactic acid formed via aerobic glycolysis was published in 2006 [220]. This study presented the following potential rationales as to the advantages that lactic acid could provide to the tumor: 1) induction of cell death in neighboring stromal cells due to necrosis or caspase-mediated apoptosis pathways thus producing potential space into which the tumor cells may proliferate; 2) promotion of angiogenesis through acid-induced release of vascular endothelial growth factor (VEGF) and IL-8; 3) promotion of extracellular matrix degradation by inducing normal cells to release proteolytic enzymes; or 4) inhibition of immune response to tumor antigens.

An extremely interesting hypothesis as to an advantage that may be provided to cancer cells by the production of lactic acid was recently proposed by Sonveaux, et al. [221]. These authors investigated two cell lines; namely, a human cervix squamous carcinoma cell line (SiHa), characterized by relatively low glucose utilization and limited lactate release, and a human colorectal adenocarcinoma cell line (WiDr), characterized by a predominantly aerobic glycolytic metabolism. Exogenous lactate could effectively replace glucose to fuel SiHa cell respiration. Of particular interest was the finding that SiHa tumors contained two viable tumor cell sub-populations defined by differential pO_2_ and MCT1 expression, an MCT isoform responsible for lactate uptake. A first subset of cells, located in the well-vascularized and oxygenated tumor margin, expressed MCT1. The other sub-population was hypoxic, poorly vascularized, and did not express MCT1. That MCT1 plays a role in metabolism of cancer cells was demonstrated by the fact that inhibition of MCT1 was found to inhibit tumor growth in a syngeneic mouse model. A rather unusual model was developed based on these findings; namely, that lactate released as the end product of glycolysis in the hypoxic tumor compartment prominently fuels the oxidative metabolism of the oxygenated tumor cell sub-population, thereby sparing glucose for oxygenated cells. Therefore, interfering with the transport of lactate by the blockade of MCT1 inhibits tumor cell proliferation.

There are also data with respect to a possible role of lactic acid in cancer patients. For example, Fischer, et al. [222] reported a positive correlation between lactate serum levels and tumor burden in 140 patients with nine different types of cancer. In order to test the possibility that at least one effect of lactic acid might be to compromise immune functions, the authors studied the effect of lactic acid on CD8+ cytotoxic T lymphocytes (CTL). They report that lactic acid inhibited CTL proliferation, up to 95%, impaired cytokine production, and inhibited the cytotoxicity of the CTLs by up to 50%. The potential immunosuppressive role of lactic acid has been recently reviewed [223], while another recent review [166] discusses the effect of increased lactic acid in a number of cancers and cancer cell lines.

Lastly, a very recent article should be mentioned which reported that tumor derived lactic acid can induce the expression of VEGF, an effect noted above, but can also induce the M2-like polarization of tumor-associated macrophages [224]. In addition, evidence was presented indicating that this effect of lactic acid is mediated by HIF-1α. Clearly these interesting results warrant further study.

In actuality, no information has been presented in this analysis that provides any additional support for the hypothesis that lactic acid production provides CCRCC cancer cells with a competitive advantage. The gene expression data are consistent with this hypothesis, however, which is not the case for the aerobic glycolytic phenotype playing a role with respect to the synthesis of key biomolecules. The role that lactic acid may be playing in cancer has recently been reviewed [225].

The strength of this study is that it provides a detailed understanding of “the context-dependent metabolic needs of cancer cells to effectively target metabolism for therapeutic benefit.” [7] Given that all of the gene expression data were derived from CCRCC cancer patients via a comparison of values in tumor tissue to adjacent normal tissue, the results are completely internally consistent. In addition, the work is focused in that all differences with respect to gene expression values relate to the extended Warburg effect network. In general GWAS identify differences in expression values with the hope of finding key genes that are linked to cancer. Although these differences are usually allocated to gene ontology pathways, it is difficult to put them into context without being able to examine downstream and upstream events that may not have exhibited a significant FC.

There are some clear limitations with respect to this study. The first, and perhaps most important, is the fact that the results are derived from only nine CCRCC patients. In order to attempt to strengthen the relationships described above, we looked at an earlier study (GSE781) reported by Lenburg et al. [226] that also provided data comparing gene expression values in nine CCRCC patients seven of which also had data on adjacent normal tissue. A hierarchical cluster analysis indicated a clear separation between gene expression values in tumor and adjacent normal tissue, as was the case for the GSE6344 data (Supplementary Figure S2). The Lenburg data set did not indicate whether calls were present, marginal or absent. Consequently, in cases where data from more than one probe for the same gene were provided, selection criteria based on this information could not be used. As a consequence, probe sets with the highest average signal intensity calculated across all samples were chosen. As indicated in the Methods section, this was the third option to select the most reliable probe set for GSE6344. The agreement between the two sets of gene expression values applied to the extended Warburg effect network was excellent, particularly when genes characterized by a large number of absent calls in data set GSE6344 are ignored. All of the data obtained from GSE781 as well as the average gene expression changes for both GSE6344 and GSE781 are shown in Supplementary Table S2.

A more recent paper appeared while the work on this analysis was well underway [227]. This very large study by the Cancer Genome Atlas Research Network obtained a complete data set from 372 CCRCC patients, including not only comparative gene expression values but mutation analyses and copy number variation as well. It would have been beyond our capabilities to have mapped the large amount of gene expression data from this study on to our extended Warburg effect network. The authors did present a brief discussion of their results with respect to glycolysis, the TCA cycle, and the PPP in the context, however, of correlation with survival. For those patients with a poorer prognosis, glycolytic genes were over-expressed, whereas genes involved in the TCA cycle were under-expressed. These results are in complete agreement with the analysis provided herein. On the other hand a number of genes in the PPP were over-expressed (*G6PD, PGLS, TALDO, TKT*), which is not in agreement with our analysis. In addition, both *ACACA* and *FASN*, two of the three genes that code for the proteins catalyzing the second and third steps of fatty acid synthesis, were over expressed in individuals with a poor prognosis. This result is also in contrast to our analysis. However, given that the nine patients we analyzed had either stage 1 or stage 2 CCRCC, it is unlikely that any of them had progressed to a stage where survival was an issue. This point illustrates another limitation of this study; namely, that the data were obtained from only stage 1 and 2 tumors. The results reported in the Cancer Genome Atlas Research Network publication [227] clearly suggest that some of our results might well have been different had gene expression values from stage 3 and 4 tumors also been available. This point was made above with respect to FASN protein expression in later stage tumors.

A third issue is that gene expression values do not necessarily correlate with protein activities. Not only is there a possibility of alternate splice varieties, a point amply discussed with respect to *PKM*, but protein activity can be significantly altered by posttranslational modifications, which cannot be determined by gene expression values. There have been a number of proteomic studies designed to investigate relevant protein activities in CCRCC, and these have been cited above. In most cases, the directional change in enzyme activity reflects the same directional change in gene expression values. As was pointed out earlier in this section, the major strength of this analysis is that it is focused on transcriptomic changes in human patients with CCRCC and compares tumor tissue data with adjacent normal tissue data. Although there is no question that proteomic, phosphoproteomic and metabolomic data would clearly provide highly valuable additional information, for it to be useful it would have to be obtained from human CCRCC patients in which tumor levels are compared to normal adjacent tissue. Unfortunately, at this time these data do not exist for the majority of the gene products investigated herein.

Gene expression values do not directly provide information on the level of important cofactors produced in many of the steps of the extended glycolytic pathway, such as ATP and NAD+. The importance of these cofactors can be seen by the fact that several articles have been published that have developed an *in silico* model of extended glycolysis in cancer. These authors utilized what they term an Objective Function; that is, the constraint that must be satisfied to maximize biomass, which includes these two cofactors as well as NAD(P)H. Such a model clearly predicts a number of the key changes in gene expression values identified herein and by other investigators [228]. Therefore, quantifying the changes in such cofactors comparing cancer tissue to normal tissue is of significant importance in understanding the role of the aerobic glycolytic phenotype in cancer.

The last issue is that in no case has a potential transcription factor been identified responsible for the increase or decrease in the expression values for the genes in the extended glycolysis network with the exception of HIF. As has been pointed out, it is well known that most of the genes that code for glycolytic proteins are under the transcriptional control of HIF-1, which is constitutively active in CCRCC due to inactivation of the VHL protein. Although almost all glycolytic genes were indeed found to be over-expressed, there are wide differences in the degree of over-expression, and transcription factors such as AKT or c-myc, or other proteins such as mTOR or p53, could be playing important roles. In all cases where an unusually large change in expression level was observed, an attempt was made to determine if the protein involved could be playing a different role than would be anticipated from the extended glycolysis pathway. However, with the exception of the role played by ALDOB in gluconeogenesis, no such alternate functions were found. As a consequence, it is possible that such unusually large changes are due to changes in levels of key transcription factors or signaling pathways.

A number of key genes have been identified that could serve as valid targets for anti-cancer pharmaceutical agents. Genes that are highly over-expressed include *ENO2, HK2, PFKP, SLC2A3, PDK1,* and *SLC16A1*. Genes that are highly under-expressed include *ALDOB, PKLR, PFKFB2, G6PC, PCK1, FBP1, PC,* and *SUCLG1*. It cannot be overemphasized that these results apply only to CCRCC and not to other cancers. It is highly likely that contextual differences exist, and it will be necessary to perform a similar analysis for other cancers that exhibit the aerobic glycolytic phenotype to identify such contextual differences.

## METHODS

### Extended Glycolysis Network

The extended glycolysis network was constructed in Cytoscape, version 2.8.2 [229] (Supplementary Figure S1). The glycolysis/gluconeogenesis network was downloaded from WikiPathways [230]. Following this, both the TCA cycle and the pentose phosphate pathway were downloaded from WikiPathways and merged with the glycolysis/gluconeogenesis pathways. The short pathways, including serine/glycine synthesis, fructose metabolism, alternate splicing of the PKM gene, glutamate utilization, and the initial steps of fatty acid synthesis, were curated from the references noted in the relevant sub-section in the Results and Discussion.

### Gene Mapping

GSE6344_Combofiles were downloaded from GEO. The authors have created these tables from the A and B chip of HG_U133 Set. The MAS5 corrected signal intensities and the absolute calls were taken and mapped to the corresponding genes from the extended glycolysis network using Affymetrix NetAffx annotations for HG-U133A and HG-U133B (version 33).

A representative probe set was selected for genes which are represented by more than one probe set. Selection criteria were:

The signal intensities for all representative probe sets and all twenty samples were used for hierarchical clustering with Euclidean distance as distance and average linkage as cluster method.

All gene names conform to HGNC nomenclature and were taken from Entrez Gene.

### Data Analysis

Fold change (FC) was calculated for all genes by dividing the signal intensity from the tumor sample by the signal intensity of the adjacent normal tissue for each patient. When one or more of both normal tissue and tumor tissue calls were absent, these were excluded from the calculation. When all calls were absent, an average FC was calculated, but the result was not assumed to be reliable as noted in the text. In cases where the gene expression level in the tumor sample was less than that of adjacent normal tissue, the FC was defined as the negative reciprocal of this value. In cases where all patients exhibited a positive FC for a particular gene, the numerical average was used to designate the average FC for that gene. In cases where there was at least one negative FC, all negative FCs were converted to positive changes by taking the reciprocal. Since these FCs are no longer normally distributed, the sum of the ln values for all FCs was calculated, and the average of the ln value was then converted to the average FC by exponentiation. If the average result was <1, the FC was represented by the negative reciprocal. Genes were identified as differentially expressed if the average FC was greater than 2 or less than −2.

P-values were calculated via Excel using the ln(FC) if the FC was positive or ln(ABS(1/FC) if the FC was negative. A 2-tailed t test method was utilized. P-values <0.05 are considered to be statistically significant. No correction for multiple testing was made.

(i) Most samples with reliable signal intensities as represented by “Present Calls”. If less than 50 % of the samples had a Present Call this probe set was marked as not reliable.
(ii) Probe sets with _x_at or s_at were discriminated compared to _at probe sets.
(iii) Probe sets with the highest average signal intensity calculated across all samples.

## SUPPLEMENTARY MATERIAL, TABLES AND FIGURES




