# Supplementary material for: Analysis and interpretation of transcriptomic data obtained from extended Warburg effect genes in patients with clear cell renal cell carcinoma
Source: Oncoscience. 2015 Feb 17;2(2):151–86. doi: 10.18632/oncoscience.128 (PMC4381708; doi:10.18632/oncoscience.128)
Supplement: Supplementary file 2 [file oncoscience-02-0151-s002.pdf]

Analysis and interpretation of transcriptomic data obtained from extended Warburg effect genes in patients with clear cell renal cell carcinoma

Supplementary Material

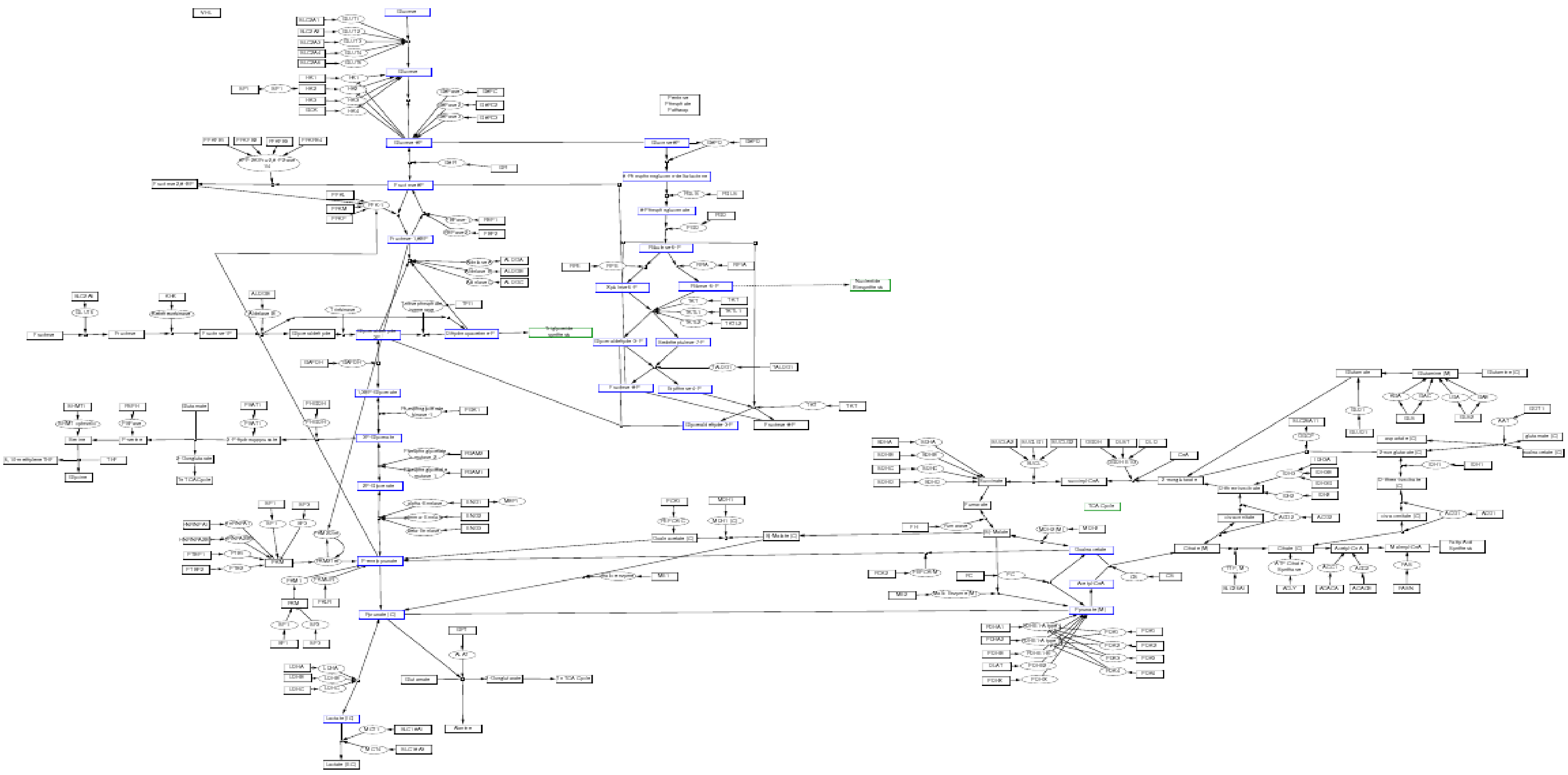

Supplementary Figure S1: Extended Warburg effect network constructed using Cytoscape. The network includes glycolysis, gluconeogenesis, the pentose phosphate pathway (PPP), the TCA cycle, the serine/glycine pathway, and partial glutaminolysis and fatty acid synthesis pathways.

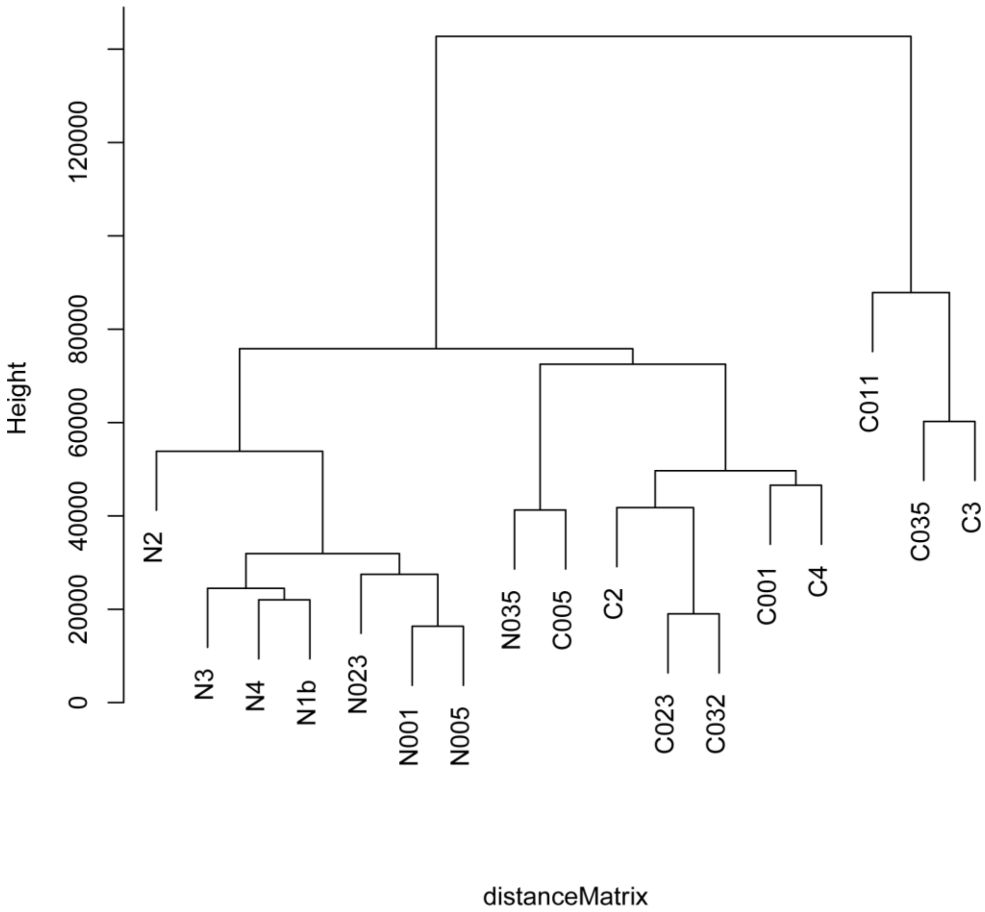

Supplementary Figure S2: Hierarchical cluster analysis. Distance matrix for the 10 CCRCC patients included in data set GSE6344 using the average linking clustering method based on Euclidean distances. Only seven of the 10 patients had data for both tumor and adjacent normal tissue.
